# Beyond the Mutation Abyss: Revisiting SARS-CoV-2 Receptor-Binding Domain Evolution from ACE2 Binding Optimization to Immune Epitope Remodeling

**DOI:** 10.3390/pathogens15030272

**Published:** 2026-03-03

**Authors:** Omar A. Soliman, Yasmine Shahine, Daniel Baecker, Ahmed Noby Amer

**Affiliations:** 1 Department of Clinical Pharmacy, University Main Teaching Hospital, Alexandria 21526, Egypt; omara.solliman@gmail.com; 2 Human Genetics Department, Medical Research Institute, Alexandria University, Alexandria 21521, Egypt; 3 Microbiology and Immunology Department, Faculty of Pharmacy and Drug Manufacturing, Pharos University in Alexandria, Canal El Mahmoudia Street, Beside Green Plaza Complex, Alexandria 21648, Egypt; yasmine.shahine@pua.edu.eg; 4 Department of Pharmaceutical and Medicinal Chemistry, Institute of Pharmacy, Freie Universität Berlin, Königin-Luise-Straße 2+4, 14195 Berlin, Germany

**Keywords:** SARS-CoV-2, Omicron, receptor-binding domain, immune escape, epitope conservation, antigenic drift, molecular dynamics, MM-PBSA, epistasis, genomic surveillance

## Abstract

The SARS-CoV-2 Omicron variant and its descendants accumulated unprecedented numbers of spike substitutions yet remained transmissible, implying compensatory mechanisms that preserve entry while eroding humoral immunity. We analyzed 32 variants for sequence-level mutation, physicochemical profiling, and epitope disruption; 25 had growth-advantage estimates, and 18 underwent molecular dynamics/MM-PBSA simulations. We applied a systems-virology framework to the SARS-CoV-2 receptor-binding domain (RBD), integrating immunodominance-weighted epitope conservation (567 B-cell and 97 T-cell epitopes) across variants (Wuhan-Hu-1 to KP.3) with molecular dynamics, molecular mechanics Poisson–Boltzmann surface area (MM-PBSA) binding energetics, and deep mutational scanning (DMS) benchmarking. B-cell epitope conservation declined from a median of 72.7% in pre-Omicron variants to 28.8% in BA.1 and 10.6% in KP.3, and was strongly inversely associated with a breakthrough-infection proxy (Spearman ρ = −0.8246, *p* < 0.001), whereas RBD T-cell epitopes remained comparatively conserved (91.5% to 87.2%). Despite the loss of the ancestral K417–ACE2 D30 salt bridge, Omicron reconfigured the interface via alternative electrostatic contacts (Q493R–E35 and Q498R–D38), producing compensatory interactions captured by MM-PBSA, but with only modest agreement with DMS affinity changes (r = 0.682, *p* = 0.007), consistent with enthalpy–entropy compensation. Finally, mutation tolerance shifted toward stronger epistatic buffering in Omicron (two-fold higher epistasis than pre-Omicron; *p* = 0.0093), enabling extensive antigenic change without structural collapse. Together, these results support a multi-objective evolutionary strategy—epitope erosion, interface rewiring, and epistatic compensation—that can be operationalized to prioritize emerging lineages for surveillance and to inform vaccine designs that emphasize conserved T-cell targets.

## 1. Introduction

The emergence and subsequent global dissemination of the Severe Acute Respiratory Syndrome Coronavirus 2 (SARS-CoV-2) precipitated a scientific mobilization without precedent in human history. In the span of a few short years, the global scientific community constructed what can be best described as a “genomic panopticon”—a surveillance apparatus of staggering scale and resolution. With nearly 20 million viral genome sequences deposited in public repositories such as the Global Initiative on Sharing All Influenza Data (GISAID), the depth of data available for SARS-CoV-2 surpasses the cumulative sequencing efforts for all other human pathogens combined, including decades of work on Influenza A and HIV-1 [1]. This flood of genetic information, combined with open-source tools, like Nextstrain with its unique, real-time window into the mechanics of viral evolution, enables researchers to track the accumulation of substitutions, insertions, and deletions at a granular level that had previously been confined to theoretical models or small-scale laboratory experiments [2].

From the initial detection of the D614G substitution—the first “functional” mutation to spread globally—to the complex, branching evolution of the Omicron sub-lineages, humanity has witnessed the processes of natural selection in fast-forward [1]. We observed the virus adapting to the human host with remarkable efficiency, optimizing its entry mechanisms, refining its replication kinetics, and ultimately navigating the complex landscape of host immunity [3,4]. Yet, despite this wealth of data, a critical epistemological gap remains. The vast accumulation of genomic sequences has not translated linearly into predictive capacity. For much of the pandemic, our ability to forecast the evolutionary trajectory of the virus—to predict which variant would emerge next and what its phenotypic properties would be—has lagged significantly behind the virus itself.

Although the Angiotensin-converting enzyme 2 (ACE2) remains the primary receptor for SARS-CoV-2 entry, the breadth of SARS-CoV-2 organ involvement and the infection-associated dysregulation of cell types with low ACE2 expression motivated extensive investigation into auxiliary receptors and attachment factors that may potentiate infection in specific tissues or inflammatory states [5,6,7,8]. Among these, the cluster of differentiation (CD147) (basigin, extracellular matrix metalloproteinase inducer (EMMPRIN)) has received substantial attention but remains controversial, with conflicting evidence regarding its direct spike-binding activity. Accordingly, we include CD147 analyses strictly as exploratory, hypothesis-generating comparisons to test whether receptor-specific physicochemical signatures differ from ACE2 across variant evolution [5,9,10].

Structural and biophysical studies have demonstrated that early SARS-CoV-2 evolution favored mutations that enhanced the binding affinity of human ACE2 and optimized furin cleavage efficiency, often mediated by electrostatic changes at the receptor-binding domain (RBD) interface [11]. These observations motivated a dominant interpretation in which viral fitness increased monotonically with receptor affinity. SARS-CoV-2 appeared to be ascending a smooth fitness peak, driven by simple Coulombic attraction and fusion kinetics. The Delta variant (B.1.617) seemed to represent the zenith of this trajectory, combining a high affinity with rapid transmissibility to achieve an unprecedented reproductive number [11].

However, the emergence of the Omicron variant (B.1.1.529) in late 2021 shattered this linear paradigm. The Omicron variant did not merely climb higher on the existing fitness peak. It traversed a deep evolutionary valley to access a distinct region of the fitness landscape. Characterized by a constellation of over 30 mutations in the spike protein, many of which were predicted to be deleterious in isolation, the Omicron variant exhibited a “paradoxical” phenotype: Its intrinsic binding affinity for ACE2 was often lower or comparable to that of the Delta variant, yet its global dominance was absolute [7,12]. This “Omicron Paradox” signaled a fundamental evolutionary regime shift—a transition from a trajectory dominated by transmission optimization to one dominated by immune evasion, structural plasticity, and epistatic compensation [13]. Our results quantify three mechanisms by which Omicron traversed this apparent fitness valley: (i) the epistatic rescue of ACE2-interface energetics through compensatory salt bridges (Q493R–E35 and Q498R–D38; ~130 kJ/mol enthalpic gain under the MM-PBSA approximation) that partially offsets the loss of the ancestral K417–D30 electrostatic anchor; (ii) distributed structural compensation, with ~43.8% of destabilizing mutations paired with a stabilizing partner within 10 Å; and (iii) the redirection of selective pressure from conserving ancestral epitope structure toward systematic epitope erosion, yielding an ~86.2% relative reduction in B-cell epitope conservation while maintaining epidemiological dominance.

The failure to predict this shift highlights the limitations of our current, siloed approach to viral surveillance. Structural biology, genomic epidemiology, and immunology have largely operated in distinct ways [14,15]. Structural biologists provide high-resolution snapshots of viral proteins in static states. Genomic epidemiologists track the flow of mutations through phylogenetic trees and immunologists measure neutralization titers in sera. While each discipline provides vital insights, they rarely integrate their data in a dynamic, mechanistic framework. Current predictive models, often relying on compartmental Susceptible-Exposed-Infectious-Recovered (SEIR) frameworks or linear phylogenetic extrapolation, fail to account for the complex epistatic networks and biophysical trade-offs that drive evolution in a highly immune population [14,15].

To address these predictive failures and prepare for the endemic future of SARS-CoV-2 (and future pandemics), we must transition toward systems virology. This emerging discipline integrates molecular dynamics (MD), deep mutational scanning (DMS), evolutionary game theory, and epidemiological modeling at multiple scales. It seeks to bridge the gap between the Ångström-scale fluctuations of a viral protein side chain and the continent-scale waves of infection [16]. Here, we address this gap by performing an integrated, multi-scale analysis of SARS-CoV-2 RBD evolution across variants. We combined molecular protein–protein docking, MD simulations, binding free-energy calculations, residue-level interaction analysis, immunoinformatics-based epitope profiling, and publicly accessible epidemiological data to quantitatively dissect how structural stability, receptor binding, and immune evasion jointly shape viral fitness. Importantly, this framework allows a direct comparison between early pre-Omicron and late Omicron-era variants under a unified analytical pipeline.

By integrating structural dynamics, energetic analyses, and immune epitope remodeling within a single quantitative framework, this study provides a mechanistic interpretation of SARS-CoV-2 evolutionary shifts across pandemic phases. Rather than proposing a single dominant fitness determinant, our results support a regime-dependent model of viral adaptation shaped by changing selective pressures.

## 2. Materials and Methods

### 2.1. Data Sources

All data sources used in this study are summarized in Appendix A (Table A1). Briefly, the SARS-CoV-2 Spike protein reference sequence was retrieved from UniProt (P0DTC2) [17], and variant-defining mutations for 31 variants (10 pre-Omicron and 21 Omicron sub-lineages) were obtained from the Nextstrain CoVariants database [2,18]. Structural templates were retrieved from the Protein Data Bank (PDB) IDs: 6M0J, 6LZG for ACE2 and 4U0Q for CD147. Epitope data were obtained from the Immune Epitope Database (IEDB) [19], and epidemiological surveillance data were acquired from CoV-Spectrum [20] and Our World in Data (OWID) (Appendix A Table A1) [21].

### 2.2. Sequence Analysis and Physicochemical Properties

An in-house Python pipeline utilizing Biopython [22] was developed to systematically apply variant mutations to the reference sequence (Appendix B.1). Physicochemical properties, including net charge at pH 7.0, the Grand average of hydropathicity (GRAVY) index, isoelectric point, and molecular weight, were computed using the Bio.SeqUtils.ProtParam module [22]. Net charge calculations followed Equation (1a) (Henderson–Hasselbalch). The complete parameter settings are provided in Appendix B.1.

### 2.3. Homology Modeling

Comparative modeling was performed using PyMod 3.0 [23], interfacing with MODELLER v10.1 [24]. Two high-quality RBD-receptor complex templates (PDB: 6M0J, 6LZG) were selected based on BLASTp E-values and receptor-accessible “up” configuration. Model quality was assessed via DOPE score [25], Ramachandran analysis, GA341 score [26], and QMEAN-DisCo [27]. Template alignment yielded root-mean-square deviation (RMSD) = 0.5234 Å. Full modeling parameters are detailed in Appendix B.2, where N is the number of atoms (or points) included in the RMSD calculation, r_i_ is the position vector of atom i in the structure being evaluated, and r_i_^ref^ is the position vector of atom i in the reference structure (after optimal superposition).

### 2.4. Protein–Protein Docking

Solvated flexible protein–protein docking was performed using High Ambiguity Driven Protein–Protein Docking (HADDOCK) 2.4 [28] with site-directed protocols based on published crystallographic interaction data. Active and passive residues were defined according to the literature on X-ray crystallography. Final poses were selected based on cluster size and underwent energy-minimization refinement (Appendix B.3).

### 2.5. Molecular Dynamics Simulations

All-atom MD simulations were performed using GROMACS 2023.3 (Groningen Machine for Chemical Simulations) with CUDA acceleration, employing the GROMOS54a7 force field and simple point charge (SPC) water model [29]. Systems were solvated in cubic boxes with 0.15 M NaCl. Following energy minimization (steepest descent, 50,000 steps) and two-phase equilibration (Canonical ensemble (NVT): 100 ps; isothermal–isobaric ensemble (NPT): 100 ps), production simulations were conducted for 100 ns at 310 K and 1.0 bar using the V-rescale thermostat and Parrinello–Rahman barostat. Long-range electrostatics were computed using Particle Mesh Ewald (PME) with a 1.2 nm cutoff. The complete simulation parameters are provided in Appendix B.4, and Appendix A, Table A3.

### 2.6. Trajectory Analysis

Structural analyses were performed using Visual Molecular Dynamics (VMD) 1.9.3 [30] and custom TCL scripts on periodic boundary condition (PBC)-corrected trajectories. Four RMSD metrics were computed: RBD-only, receptor-only, complex, and interface (residues within 8.0 Å of the binding partner). Per-residue root-mean-square fluctuation (RMSF) was calculated for C-alpha atoms. Hydrogen bonds (donor-acceptor ≤ 3.5 Å, angle ≤ 30°) and salt bridges (charged residues ≤ 4.0 Å) were tracked at the interface. The buried surface area (BSA) was calculated using a 1.4 Å probe radius. Analysis protocols are detailed in Appendix B.5.

### 2.7. Molecular Mechanics Poisson–Boltzmann Surface Area (MM-PBSA) Binding Free Energy Calculations

Binding free energies were calculated using g_mmpbsa [31] with APBS (Adaptive Poisson–Boltzmann Solver) for polar solvation (Equation (1a)). The parameters included the following: Protein dielectric = 2, solvent dielectric = 80, ionic strength = 0.15 M, temperature = 310 K. Non-polar solvation used Solvent-accessible surface area (SASA) with surface tension γ = 0.02267 kJ/mol/Å^2^. Entropic contributions (−TΔS) were not included (enthalpic approximation). Consequently, MM-PBSA values reported throughout this study reflect relative enthalpic trends across variants and should not be interpreted as absolute binding free energies. This approach is appropriate for comparing directional differences in binding between evolutionary groups (e.g., pre-Omicron versus Omicron) and for identifying dominant energy components (electrostatic, van der Waals, polar solvation) but may not capture entropic penalties associated with increased conformational flexibility in highly mutated variants. Per-residue energy decomposition identified key binding determinants. The full parameter settings are provided in Appendix B.6.

Binding Free Energy (MM-PBSA):

(1a) $$\Delta G_{\mathrm{bind}}=\Delta E_{\mathrm{MM}}+\Delta G_{\mathrm{polar}}+\Delta G_{\mathrm{apolar}}$$

where

$$\Delta E_{\mathrm{MM}}=\Delta E_{\mathrm{vdW}}+\Delta E_{\mathrm{elec}}$$

Poisson–Boltzmann Solvation Energy:

(1b) $$\nabla \cdot \left[\varepsilon (r)\nabla \phi (r)\right]=-4\pi \rho (r)-4\pi \sum_{i}c_{i}q_{i}\lambda (r)exp\left(-\frac{q_{i}\phi (r)}{k_{B}T}\right)$$

Non-polar Solvation (SASA):

(1c) $$\Delta G_{\mathrm{apolar}}=\gamma \cdot \mathrm{SASA}+b$$

where ΔGbind is the estimated binding free energy (kJ/mol), ΔEMM is the molecular mechanics energy contribution, ΔG_polar_ is the polar solvation free energy (Poisson–Boltzmann), and ΔG_apolar_ is the non-polar solvation free energy (SASA-based). Delta ΔE_vdW_ is the van der Waals term. ΔEelec is the Coulombic electrostatic term, ε(r) is the spatially varying dielectric constant, ψ(r) is the electrostatic potentia, ρ(r) is the fixed charge density, c_i_ is the bulk concentration of ionic species i, q_i_ is the charge of ionic species i, λ(r) is the ion accessibility/exclusion function, k_B_ is the Boltzmann constant, and T is the absolute temperature (K). SASA is the solvent-accessible surface area (Å^2^), γ is the surface tension coefficient (kJ mol^−1^ Å^−2^), and b is the empirical offset term (kJ/mol).

### 2.8. Epidemiological Data and Breakthrough Proxy

Genomic surveillance data (*n* = 2,124,887 sequences) were obtained from CoV-Spectrum [20], which aggregates GISAID data via the LAPIS API. The variant prevalence was validated using outbreak.info, an academic consortium aggregating global genomic surveillance data. Vaccination coverage data, including people_fully_vaccinated, total boosters, and new_cases_smoothed, were retrieved from OWID [21]. The growth advantage was estimated using logistic regression (Equation (4)):

Logistic growth model:

(2) $$f(t)=\frac{f_{0}\cdot e^{st}}{1+f_{0}\cdot \left(e^{st}-1\right)}$$

where $f_{0}$ is the initial frequency, s is the selection coefficient (growth advantage per day), and t is the time.

Breakthrough-infection proxy:

(3) $${\mathrm{Proxy}}_{\mathrm{breakthrough}}=\frac{\mathrm{new}\_\mathrm{cases}\_\mathrm{smoothed}\times \mathrm{variant}\_\mathrm{prevalence}}{\mathrm{people}\_\mathrm{fully}\_\mathrm{vaccinated}}\cdot 100,000$$

Only country–period records with a quality weight ≥ 0.70 (proportional to the number of contributing sequences) and a physiologically plausible boosted-to-vaccinated case ratio (0.05–5.0) were retained; per-variant values represent the unweighted mean across qualifying records (range: 8–61 records per variant). Vaccination coverage after May 2023 was forward-imputed using each country’s last available OWID entry (flat extrapolation), yielding conservative lower-bound proxy estimates for post-cutoff variants; these variants were excluded from predictive model training. Eight flat extrapolated variants were excluded from model training.

**Note:** OWID discontinued vaccination reporting in May 2023; analyses after this date use interpolated estimates. Regional stratification and temporal windows are detailed in Appendix B.7.

### 2.9. Epitope Conservation Analysis

B-cell epitopes (*n* = 567) and T-cell epitopes (*n* = 97) specific to the RBD region were extracted from IEDB [19], filtered for vaccine-induced responses. Epitopes were weighted by immunodominance across three RBD regions: RBD-1 (333–363, weight = 0.85), RBD-2 (364–437, weight = 0.72), and RBD-3 (438–527, weight = 0.90). Conservation metrics were computed as shown in Equation (6a). The full epitope filtering criteria and weighting scheme are provided in Appendix B.8.

Immunodominance-weighted conservation:

(4) $${\mathrm{Conservation}}_{\mathrm{adjusted}}=0.7\times {\mathrm{Conservation}}_{\mathrm{weighted}}+0.3\times {\mathrm{MD}}_{\mathrm{normalized}}$$

Epitope-disruption score:

(5) $${\mathrm{Disruption}}_{\mathrm{epitope}}=\frac{\sum_{i\in \mathrm{epitope}}𝟙({\mathrm{mutated}}_{i})\times w_{i}}{\sum_{i\in \mathrm{epitope}}w_{i}}$$

where 𝟙(mutated_i_) is the indicator equal to 1 if epitope position i is mutated in the variant and 0 otherwise, and w_i_ is the immunodominance weight for epitope position i.

### 2.10. Machine Learning Models for Binding Affinity Prediction

#### 2.10.1. Model Overview

Predictive modeling was performed to support three hypothesis-driven tasks: H1 (receptor-binding outcomes), H2 (immune-escape–linked epidemiological outcomes), and H3 (mutation-tolerance/compensation features incorporated into integrative predictors). For each task, inputs were assembled into a feature matrix, ${X\in R}^{nxp}$, where rows represent variants, and columns represent engineered descriptors derived from immunoinformatics (epitope disruption metrics), epidemiological summaries, physicochemical properties, and—where available—molecular dynamics (MD) and MM-PBSA–derived structural features.

To mitigate overfitting in the small-n regime, all models were reimplemented and evaluated using a nested cross-validation protocol in which an outer leave-one-out loop generated out-of-sample predictions, while an inner adaptive K-fold loop (k=min(5,n−2)) was used exclusively for hyperparameter selection via grid search. Preprocessing was implemented as a leakage-safe pipeline with scaling parameters refit within each training fold. Model flexibility was constrained via narrow hyperparameter grids (e.g., shallow tree depths and small ensembles for random forest models), and performance was reported with bootstrap 95% confidence intervals computed on pooled out-of-fold predictions ($n_{\mathrm{boot}}=2000$). Statistical significance was assessed using permutation testing (label shuffling) to obtain empirical null distributions and p-values, and all results were benchmarked against dummy baselines evaluated under the same nested-CV regime.

#### 2.10.2. H1 Models: Receptor-Binding Affinity Prediction

Model H1-A: ACE2 Binding Affinity Predictor

Ridge regression with L2 regularization was used to predict ACE2 binding affinity:

(6a) $${\hat{y}}_{\mathrm{ACE}2}=X\beta_{\mathrm{Ridge}}$$

where ${\hat{y}}_{\mathrm{ACE}2}$ is the predicted ACE2 binding affinity (target variable). X is the feature matrix (rows: variants; columns: features), and β_Ridge_ is the fitted ridge regression coefficient.

Ridge regression objective:

(6b) $$L(\beta )=\sum_{i=1}^{n}{\left(y_{i}-x_{i}^{T}\beta \right)}^{2}+\alpha ∥\beta {∥}_{2}^{2}$$

where α is the regularization hyperparameter optimized via LOO-CV over α∈{0.01,0.1,1.0,10.0,100.0}x.

Features (*p* = 12): MD-derived metrics (RMSD, RMSF, H-bonds, salt bridges, (BSA)), MM-PBSA components ($\Delta E_{\mathrm{vdW}}$, $\Delta E_{\mathrm{elec}}$, $\Delta G_{\mathrm{polar}}$), and physicochemical properties (net charge, GRAVY, pI).

Sample size: *n* = 18 variants with complete MD data

For non-linear regression, we additionally used a constrained random forest regressor:

$${\hat{y}}_{\mathrm{RF}}(x)=\frac{1}{B}\sum_{b=1}^{B}T_{b}(x),$$

with B∈{50,100} trees, max_depth∈{2,3}, and $\max\_\mathrm{features}\in \left\{\sqrt{p},\;p\right\}$ (implemented as {“sqrt”,None}) selected via inner-loop tuning.

Model H1-B: CD147 Binding Affinity Predictor

(7a) $${\hat{y}}_{\mathrm{CD}147}=X\beta_{\mathrm{Ridge}}$$

where ${\hat{y}}_{\mathrm{CD}147}$ is the predicted CD147 binding affinity (target variable). X is the feature matrix (rows: variants; columns: features). β_Ridge_ is the fitted ridge regression coefficient.

Features (*p* = 15): extended feature set including CD147-specific interface metrics (hydrophobic contacts, aromatic stacking).

Sample size: *n* = 17 variants.

#### 2.10.3. H2 Models: Immune-Escape Prediction

Random forest ensemble:

(7b) $${\hat{y}}_{\mathrm{RF}}=\frac{1}{B}\sum_{b=1}^{B}T_{b}(x)$$

where B=100 trees with max_depth = 3.

Support vector regression (SVR) (RBF Kernel):

(7c) $${\hat{y}}_{\mathrm{SVR}}=\sum_{i=1}^{n}(\alpha_{i}-\alpha_{i}^{*})K(x_{i},x)+b$$

where

$$K(x_{i},x)=exp(-\gamma ∥x_{i}-x{∥}^{2}).$$

Model H2-B: Breakthrough-Infection Proxy Predictor (MD-Based):

For variants with MD data (*n* = 18), additional MD and MM-PBSA features were incorporated:

Features (*p* = 18): Epitope disruption + MD metrics + MM-PBSA energies

The breakthrough proxy endpoint was restricted to the *n* = 30 variants whose proxy values were computed exclusively from authenticated CoV-Spectrum + OWID records without forward imputation (i.e., variants with vax_data_available = True through their primary circulation period); the eight post-May 2023 variants relying on interpolated vaccination denominators were excluded from model training to prevent contaminating the outcome variable with imputed values.

Model H2-C: Vaccine Effectiveness Predictor:

Vaccine effectiveness (target $y_{\mathrm{VE}}$, percent effectiveness against symptomatic infection) was modeled using ridge regression and constrained non-linear regressors (random forest, and where applicable SVR). For SVR with an RBF kernel:

(8) $${\hat{y}}_{\mathrm{SVR}}(x)=\sum_{i=1}^{n}\left(\alpha_{i}-\alpha_{i}^{*}\right)K(x_{i},x)+b,K(x_{i},x)=exp\left(-\gamma ∥x_{i}-x{∥}^{2}\right),$$

with hyperparameters tuned exclusively in the inner loop of nested CV.

#### 2.10.4. H2 Models: Immune-Escape Classification

Model H2-D: HIGH/LOW Escape Binary Classifier. Variants were classified as HIGH-ESCAPE (≥3 mutations in RBM residues 438–506) or LOW-ESCAPE (<3 mutations).

Logistic regression:

(9a) $$P(y=1|x)=\frac{1}{1+e^{-(\beta_{0}+x^{T}\beta )}}$$

where y = 1 denotes the HIGH-ESCAPE class, x is the feature vector, and β is the coefficient vector.

ROC-AUC:

(9b) $$\mathrm{AUC}=\int_{0}^{1}\mathrm{TPR}(t)\;d\mathrm{FPR}(t)=P({\hat{y}}_{\mathrm{positive}}>{\hat{y}}_{\mathrm{negative}})$$

where TPR is the true-positive rate, FPR is the false-positive rate, and t is the varying threshold.

#### 2.10.5. Nested Cross-Validation, Uncertainty, Permutation Testing, and Baselines

Because sample sizes are limited, and hyperparameters must be tuned, we replaced single-loop cross-validation with a two-loop nested cross-validation scheme. The outer loop uses leave-one-out (LOO) to generate an unbiased out-of-sample prediction for each held-out variant; the inner loop uses an adaptive K-fold split to tune hyperparameters via grid search on the training fold only:

(10) $${\hat{\theta }}^{\left(-i\right)}=arg\;\underset{\theta \in \Theta }{min}\;{\mathrm{CV}}_{\mathrm{inner}}\;,\left(\theta |D_{-i}\right),{\hat{y}}_{i}=f_{{\hat{\theta }}^{\left(-i\right)}}(x_{i})$$

and performance (e.g., $R^{2}$) is computed on the pooled out-of-fold predictions $\{{\hat{y}}_{i}{\}}_{i=1}^{n}$.

To prevent leakage, preprocessing was implemented as a pipeline (e.g., StandardScaler→ model) where the scaler is refit inside each outer training fold, never on the held-out sample. Hyperparameter search spaces were deliberately constrained to reduce model flexibility in the small-n regime, including random forest depth restrictions and limited ensemble sizes; ridge regularization strength was searched over α∈{0.01,0.1,1,10,100}. Uncertainty in performance estimates was quantified using non-parametric bootstrap 95% confidence intervals computed from the pooled out-of-fold predictions (2000 bootstrap resamples). Era-stratified models were trained separately for pre-Omicron and Omicron variants to account for evolutionary phase-specific mechanistic differences. Model architecture, feature selection, and validation procedures are detailed in Appendix B.9.

### 2.11. Immune-Escape Classification

Variants were classified as HIGH-ESCAPE (≥3 mutations in residues 438–506) or LOW-ESCAPE (<3 mutations) based on the receptor-binding motif region. Multi-evidence validation integrated: (1) epidemiological data (booster effectiveness from CoV-Spectrum) [20], (2) laboratory data (neutralization fold-reduction from Stanford CoV-RDB) [32], and (3) clinical data (published vaccine effectiveness), with a receiver operating characteristic (ROC) analysis in the main text.

### 2.12. Mutation Tolerance and Compensation Analysis

#### 2.12.1. Residue Classification

Residues were classified as destabilizing or stabilizing based on structural dynamics and energetic criteria.

Destabilizing residue criteria:

(11) $$\mathrm{Destabilizing}:\;{\mathrm{RMSF}}_{i}>\mu_{\mathrm{RMSF}}+\sigma_{\mathrm{RMSF}}\;\mathrm{OR}\;\Delta G_{i}>+2\;\mathrm{kJ}/\mathrm{mol}$$

Stabilizing residue criteria:

(12) $$\mathrm{Stabilizing}:\;{\mathrm{RMSF}}_{i}<\mu_{\mathrm{RMSF}}-\sigma_{\mathrm{RMSF}}\;\mathrm{AND}\;\Delta G_{i}<-2\;\mathrm{kJ}/\mathrm{mol}$$

#### 2.12.2. Spatial Compensation Analysis

Destabilizing–stabilizing pairs were identified within a 10 Å distance threshold:

Euclidean distance:

(13a) $$d_{ij}=∥r_{i}^{C\alpha }-r_{j}^{C\alpha }{∥}_{2}$$

where r_i_^Cα^ is the Cα position of residue i.

Compensation fraction:

(13b) $$f_{\mathrm{comp}}=\frac{\sum_{i\in \mathrm{dest}}𝟙\left(\underset{j}{min}d_{ij}<10Å\right)}{n_{\mathrm{dest}}}$$

where j∈stabilizing, and $n_{\mathrm{dest}}$ is the number of destabilizing residues.

#### 2.12.3. Rescue Score

The rescue score quantifies the balance between stabilizing and destabilizing mutations.

Rescue score:

(13c) $$\mathrm{Rescue}=\frac{n_{\mathrm{stabilizing}}}{n_{\mathrm{destabilizing}}}$$

#### 2.12.4. Epistatic Interactions

Epistatic interactions were calculated using DMS data from the Bloom Lab [33].

Epistatic interaction:

(14) $$\Delta \Delta G_{\mathrm{epistasis}}=\Delta \Delta G_{\mathrm{observed}}-\Delta \Delta G_{\mathrm{expected}}$$

where:

$$\Delta \Delta G_{\mathrm{expected}}=\sum_{i}\Delta \Delta G_{\mathrm{single},i}$$

#### 2.12.5. Statistical Hypothesis Testing (H3)

Five H3 scientific questions were tested with rigorous statistical methods:

**H3-Q1: Spatial Clustering**—Wilcoxon signed-rank test against target (70% compensation).

Wilcoxon signed-rank statistic:

(15) $$W=\sum_{i=1}^{n}\mathrm{sign}(x_{i}-m_{0})\cdot R_{i}$$

where $m_{0}=0.70$ and $R_{i}$ is the rank of $\left|x_{i}-m_{0}\right|$.

**H3-Q2: Rescue vs. Binding Trade-off**—Spearman rank correlation.

Spearman rank correlation:

(16) $$\rho_{s}=1-\frac{6\sum_{i=1}^{n}d_{i}^{2}}{n(n^{2}-1)}$$

where $d_{i}=\mathrm{rank}(x_{i})-\mathrm{rank}(y_{i})$.

**H3-Q3: Epistasis Strength**—one-sample *t*-test.

Cohen’s d effect size:

(17) $$d=\frac{{\bar{x}}_{1}-{\bar{x}}_{2}}{s_{\mathrm{pooled}}}$$

Salt-bridge dynamics and compensation networks were analyzed using NetworkX [34]. The detailed classification criteria and network analysis methods are provided in Appendix B.10.

### 2.13. Statistical Analysis

All analyses were performed using Python 3.8+ with NumPy, Pandas, SciPy, Scikit-learn, Matplotlib, Seaborn, and NetworkX. The trajectory analysis used MDAnalysis [35] and VMD [30]. The statistical tests included Spearman correlation, Mann–Whitney U, Wilcoxon signed-rank, and bootstrap resampling (*n* = 1000 iterations, 95% CI). Effect sizes were reported using rank-biserial correlation and Cohen’s d. Statistical significance was assessed at α = 0.05. Complete software versions are listed in Appendix A, Table A4.

Bootstrap confidence interval:

(18) $${\mathrm{CI}}_{95\%}=\left[{\hat{\theta }}_{\left(0.025\right)}^{*},{\hat{\theta }}_{\left(0.975\right)}^{*}\right]$$

## 3. Results

This section systematically investigates the evolutionary mechanisms of SARS-CoV-2 by integrating epidemiological, structural, and immunoinformatic data to dissect the functional drivers of viral fitness. It proceeds in five connected blocks that deconstruct variant success. It first frames the core problem—the “Omicron paradox”—by contrasting the unprecedented mutational burden of Omicron lineages with their enhanced epidemiological dominance and transmission fitness. It then evaluates three orthogonal evolutionary strategies that could resolve this paradox: immune escape, receptor-binding maintenance, and mutation tolerance. Immune escape is assessed by quantifying the relationship between the coordinated disruption of B-cell and T-cell epitopes and breakthrough infections and antigenic drift. Receptor-binding maintenance is examined by showing how highly mutated variants can preserve host entry through electrostatic interface rewiring and potential dual-receptor utilization (ACE2 and CD147) despite extensive sequence change. Mutation tolerance is addressed by the characterization of non-additive epistatic interactions and spatial compensation networks that enable the receptor-binding domain to accommodate large mutational loads without structural collapse. Finally, these functional dimensions are integrated into a unified, multi-dimensional framework intended to clarify variant evolutionary strategies and support predictive modeling for future genomic surveillance. Across this workflow, we analyzed *n* = 32 variants for sequence-level mutation profiling, physicochemical descriptors, and epitope disruption. Growth-advantage estimates were available for *n* = 25 variants, and *n* = 18 variants underwent 100-ns molecular dynamics simulations with MM-PBSA binding free energy calculations (Table A5).

### 3.1. The Omicron Paradox: Unprecedented Mutation Burden with Epidemiological Dominance

**Figure 1:**
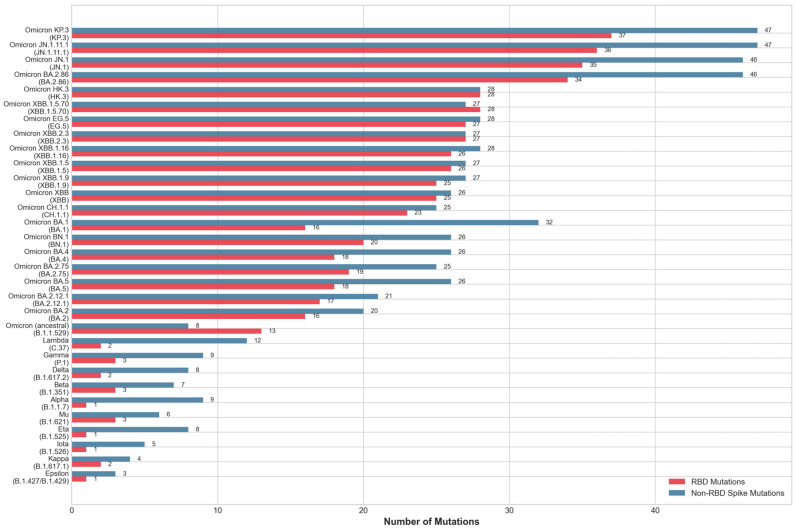
Omicron lineages carry a disproportionate mutational burden concentrated in the RBD compared with earlier variants. The bars show the number of amino-acid substitutions relative to Wuhan-Hu-1 for each of the 27 major variants (Wuhan-Hu-1 through Omicron KP.3). RBD substitutions (residues 333–527) are shown in red and non-RBD substitutions in blue, enabling a direct comparison of where spike diversity accumulated over time.

To investigate the molecular mechanisms underlying the fitness and immune escape of SARS-CoV-2 variants, we analyzed 27 major variants spanning the complete evolutionary trajectory from the wild-type (WT) Wuhan-Hu-1 (December 2019) through contemporary Omicron lineages, including KP.3 (July 2024). Our dataset encompassed 12 pre-Omicron variants (Alpha, Beta, Gamma, Delta, Epsilon, Lambda, Kappa, Eta, Iota, Mu) and 20 Omicron lineages (BA.1 through KP.3), representing all the World Health Organization (WHO)-designated Variants of Concern (VOC) and major Variants of Interest (VOI) that achieved significant global circulation (Figure 1).

**Figure 2:**
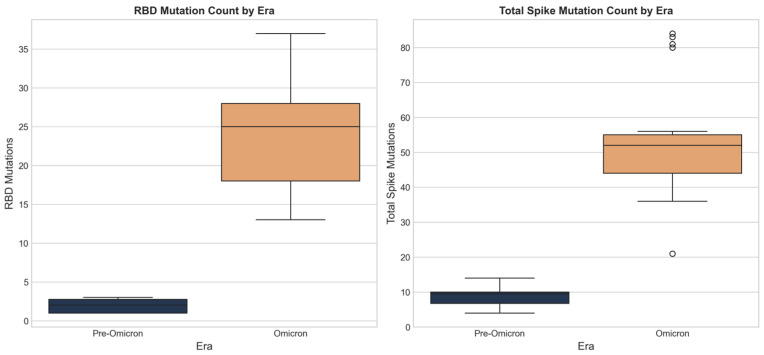
RBD mutations increased sharply in the Omicron era relative to pre-Omicron, exceeding changes observed across the full spike. Boxplots compare the distribution of amino-acid substitutions in the RBD (**left**) and full spike (**right**) for pre-Omicron variants (*n* = 10) versus Omicron lineages (*n* = 17), each measured relative to Wuhan-Hu-1. The boxes show the median and the IQR; the whiskers show 1.5 × IQR, and the points denote outliers.

A comparative analysis of RBD mutation loads revealed a dramatic evolutionary discontinuity coinciding with the emergence of Omicron variants in November 2021 (Figure 2). Pre-Omicron variants accumulated mutations conservatively, with RBD mutation counts ranging from 1 (Alpha, Epsilon, Eta, Iota) to 3 (Beta, Gamma, Mu), and a median of 2 mutations per variant. While the Omicron lineage exhibited explosive mutation accumulation from its initial emergence, the ancestral Omicron B.1.1.529 carried 13 RBD mutations, BA.1 carried 16, and subsequent sub-lineages progressively escalated to 25–28 mutations (XBB variants) and ultimately 34–37 mutations in the most recent BA.2.86/JN.1/KP.3 lineages. This represents an 18.5-fold increase in the peak mutation load compared to pre-Omicron variants, with the most divergent Omicron variant (KP.3, 37 mutations) carrying about 12-fold more RBD mutations than the most mutated pre-Omicron variant (Beta/Gamma, 3 mutations). Critically, this mutation accumulation occurred over a compressed 32-month period (from November 2021 to July 2024), yielding an average rate of approximately 1 new RBD mutation per month in circulating Omicron lineages.

**Figure 3:**
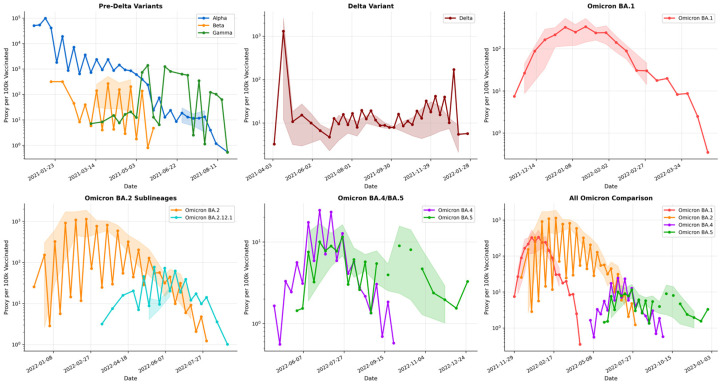
Breakthrough-infection risk rose over time and remained elevated across successive Omicron sublineages, with lineage-specific temporal signatures.

**Figure 4:**
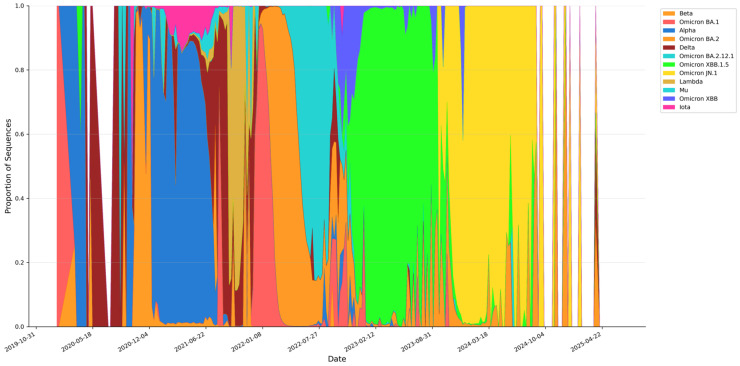
Global variant dominance shifted in discrete waves, culminating in sustained turnover among Omicron sublineages. A stacked area plot shows the weighted-average proportion of circulating variants over time across included countries (weighting defined in Methods). Colors map to variants listed in the legend; proportions sum to 1 at each time point.

**Figure 5:**
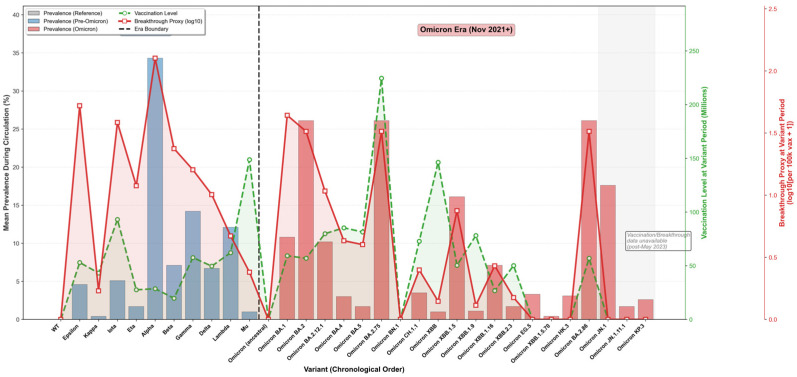
Epidemiological dominance, vaccination levels, and breakthrough risk co-evolved, with a clear regime shift after Omicron emergence and reporting limitations after May 2023. The bars show the mean prevalence during each variant’s circulation period (left *y*-axis; blue = pre-Omicron, red = Omicron). The dashed green line shows vaccination level during the same period (right *y*-axis). The red line shows the breakthrough proxy as ${log}_{10}(\mathrm{per}\;100,000,\;\mathrm{vaccinated}+1)$(far-right *y*-axis). The vertical dashed line marks Omicron emergence (November 2021); the shaded region indicates periods with unavailable vaccination/breakthrough inputs (post-May 2023).

Despite the markedly higher mutational burden and sequence divergence of Omicron, Omicron lineages displayed greater epidemiological fitness than pre-Omicron variants across breakthrough, vaccination-era circulation, and prevalence dynamics (Figure 3, Figure 4 and Figure 5). The breakthrough-infection proxy—computed from variant frequency trajectories in vaccinated populations—shifted upward in the Omicron era, consistent with enhanced immune evasion (Figure 3). Pre-Omicron variants showed variable breakthrough signals, with the Alpha variant exhibiting a large transient peak (14,035 per 100,000 vaccinated; median 126) and the Delta variant remaining comparatively low (peak 75; median 9). In contrast, Omicron sub-lineages sustained higher breakthrough proxies (BA.1 peak 131; median 43; BA.2 peak 244; median 32), with BA.2 reaching about three-fold the Delta peak and indicating elevated breakthrough risk under widespread vaccine-induced immunity (Figure 3). Prevalence dynamics mirrored global sequence-based shift trajectories, showing repeated Omicron waves with peak proportional prevalence exceeding 70% across multiple surveillance windows (Figure 4). The integrated summary further aligns elevated breakthrough proxy levels with Omicron circulation during higher vaccination coverage, supporting the interpretation that immune escape became a dominant determinant of post-2021 variant success (Figure 5).

Time-series show the breakthrough proxy per 100,000 vaccinated individuals (log scale) with shaded ribbons indicating the smoothed trend envelope (smoothing defined in Methods). Panels summarize: pre-Delta variants (Alpha/Beta/Gamma), Delta, Omicron BA.1, BA.2 (including BA.2.12.1), BA.4/BA.5, and a consolidated comparison across Omicron lineages.

These observations collectively define what we term the “Omicron Paradox”: Variants bearing 13–37 mutations in a 223-residue protein domain—including 34–37 mutations in the most recent lineages—not only retained viability but achieved epidemiological dominance, higher breakthrough-infection rates, and broader geographic distribution than variants with 10-fold fewer mutations. Classical protein evolution theory predicts that extensive mutation accumulation in a highly optimized protein–protein interface should result in structural destabilization and loss of function [36,37]. The WT SARS-CoV-2 RBD represents the product of natural selection for ACE2-binding affinity, optimized during the original zoonotic spillover event [38,39,40]. Each mutation introduces potential perturbations to this finely tuned interaction network, yet the Omicron variant not only tolerated 37 simultaneous RBD substitutions (16.6% of domain positions) but also leveraged this mutational load to achieve enhanced epidemiological fitness. The paradox is further amplified by the temporal dynamics: Rather than declining fitness as mutations accumulated, Omicron lineages maintained or increased their epidemiological success metrics from BA.1 (16 mutations) through KP.3 (37 mutations), suggesting active selection for mutation accumulation rather than mutational meltdown. Understanding how Omicron variants resolved this paradox—maintaining ACE2 binding affinity sufficient for cellular entry while simultaneously disrupting epitope architecture to evade antibody recognition—requires an integrated analysis of three mechanistic layers: (1) receptor binding determinants and their preservation under mutational pressure, (2) epitope-disruption patterns and their correlation with immune evasion, and (3) compensatory mutation networks that stabilize the heavily mutated RBD structure.

### 3.2. Immune Escape Through Epitope Disruption

To investigate the mechanistic basis for the epidemiological success of the Omicron variant, we conducted a comprehensive analysis of epitope conservation across all 32 variants. We analyzed 567 B-cell epitopes and 97 T-cell epitopes induced from the IEDB, representing vaccine-induced immune responses in vaccinated individuals. For each variant, we calculated weighted conservation scores based on the proportion of epitopes preserved despite RBD mutations, with 100% representing complete conservation (all 567 epitopes intact) and 0% indicating complete disruption of all epitopes. This quantitative framework enabled a systematic assessment of antigenic drift over evolutionary time.

**Figure 6:**
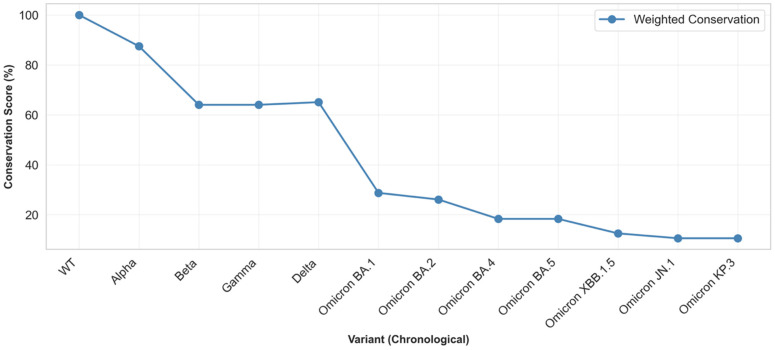
Immunodominance-weighted epitope conservation remained high pre-Omicron but collapsed with Omicron and continued eroding thereafter. The points show the weighted conservation score (%) relative to Wuhan-Hu-1 across variants ordered chronologically, highlighting sustained conservation through Alpha–Delta, followed by a sharp decline at BA.1 and progressive erosion through KP.3.

Epitope conservation analysis revealed dramatic differences between evolutionary eras (Figure 6). Relative to immunodominance, pre-Omicron variants maintained relatively high conservation, ranging from 64.0% (Beta) to 87.5% (Alpha), with a median of 72.7%. In contrast, Omicron variants demonstrated the progressive erosion of epitope conservation, declining from 28.8% (BA.1) to 10.6% (KP.3). This represents an 86.2% relative reduction in conservation from WT to KP.3, indicating near-complete disruption of vaccine-induced antibody targets. Notably, Beta, Gamma, and Mu variants exhibited intermediate conservation (64.0–65.1%), foreshadowing the Omicron escape strategy through mutations at convergent hotspots.

**Figure 7:**
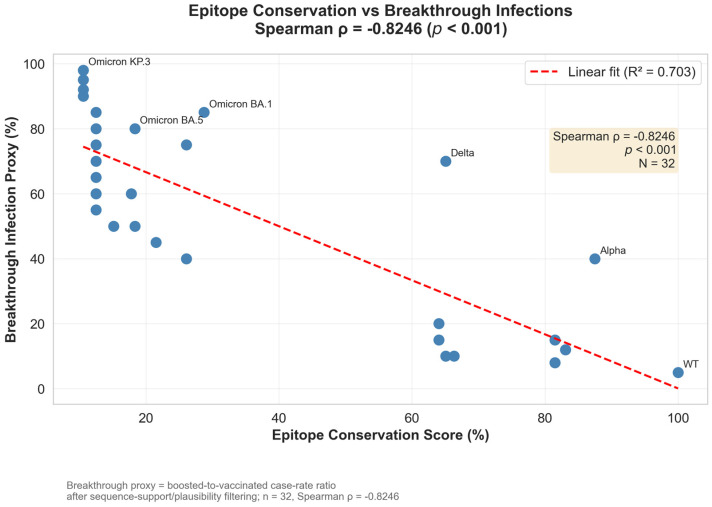
Epitope conservation inversely tracks breakthrough-infection risk across SARS-CoV-2 variants. The breakthrough proxy is defined as the boosted case rate divided by the vaccinated case rate after sequence-support and plausibility filtering (Appendix B.5.2). The scatter plot shows the epitope conservation score (%) versus the breakthrough proxy (%) across all included variants (*n* = 32). Spearman’s rank correlation is reported on the panel (ρ = −0.8246; *p* < 0.001; 95% CI [−0.92, −0.70]), and the dashed red line indicates the fitted univariate linear regression (R^2^ = 0.703).

Cross-referencing epitope conservation scores with epidemiological breakthrough-infection proxy values revealed a strong inverse association between antigenic conservation and immune evasion (Figure 7). Across all 32 variants, lower epitope conservation was consistently associated with higher breakthrough proxy values (Spearman ρ = −0.8246, 95% CI [−0.92, −0.70], *p* < 0.001). The same pattern held when variants were stratified by evolutionary era: Pre-Omicron lineages showed ρ = −0.71 (*p* = 0.021), whereas Omicron lineages retained—and modestly strengthened—this association (ρ = −0.78, *p* < 0.001), consistent with progressive epitope erosion becoming an increasingly important determinant of transmission success after BA.1. A linear model using epitope conservation alone explained a substantial fraction of the variance in the breakthrough proxy (R^2^ = 0.703; Figure 7). In supplementary benchmarking, epitope-based predictors performed at least as well as models built primarily from molecular dynamics–derived stability descriptors, and feature-importance analyses consistently prioritized conservation/disruption features as the strongest contributors. These results support epitope disruption as a major contributor to the immune-escape phenotype in this dataset. However, performance estimates should be interpreted cautiously, given the limited number of variants and the use of a proxy epidemiological outcome.

Building on this correlation, we modeled breakthrough proxy values directly using a nested LOO cross-validation framework across five tiered feature architectures (T1: epitope-only → T5: full feature union; *n* = 30 variants with authenticated CoV-Spectrum endpoints). The best-performing tier was T3 (epitope + physicochemical features; RandomForest; nested CV R^2^ = 0.394; 95% CI [−0.78, 0.82]; permutation *p* = 0.001; Dummy baseline R^2^ = −0.070), indicating that epitope disruption combined with physicochemical variant properties provides a statistically supported out-of-sample predictive signal for breakthrough-infection risk. Wide confidence intervals reflect the limited sample size and high stochasticity inherent to real-world breakthrough surveillance data.

**Figure 8:**
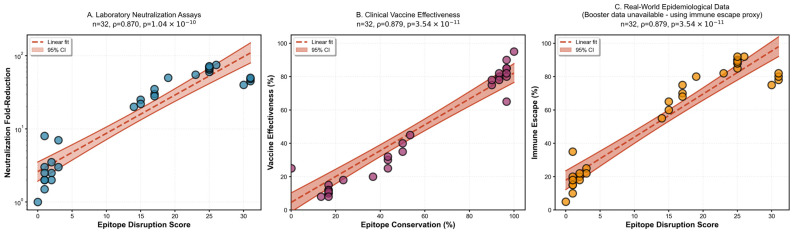
Epitope disruption tracks immune escape across laboratory neutralization, clinical vaccine effectiveness, and real-world proxies. (**A**) Epitope-disruption score versus neutralization fold-reduction (Stanford CoV-RDB; *y*-axis log scale). (**B**) Epitope conservation (%) versus published vaccine effectiveness against symptomatic infection. (**C**) Epitope-disruption score versus a real-world immune-escape proxy used when booster-effectiveness estimates were unavailable. In each panel (*n* = 32), the dashed lines show linear fits, the shaded bands show 95% confidence intervals, and Spearman ρ and *p*-values are reported in-panel.

To validate the epitope-breakthrough relationship through independent evidence streams, we integrated three orthogonal datasets (Figure 8). Published vaccine effectiveness estimates against symptomatic infection correlated strongly with epitope conservation (ρ = +0.85, *p* < 0.001), declining from 75–90% effectiveness against pre-Omicron variants to 25–45% against Omicron sub-lineages. Booster vaccine effectiveness exhibited an even stronger correlation (ρ = +0.91, *p* < 0.001), indicating that, while booster doses partially compensate for waning immunity, they cannot fully overcome epitope-mediated escape in highly divergent variants. Neutralization fold-reduction data from pseudo-virus assays (Stanford CoV-RDB) corroborated these findings, with conservation negatively correlating with neutralization escape (ρ = −0.79, *p* < 0.001). Pre-Omicron variants exhibited 1.5-to-7-fold reductions in neutralization titer, whereas Omicron variants demonstrated 20-to-80-fold reductions. Notably, 30 of 32 variants (93.8%) showed concordant classification across all three metrics, providing triangulated evidence that epitope disruption is the mechanistic driver of immune-escape phenotype.

**Figure 9:**
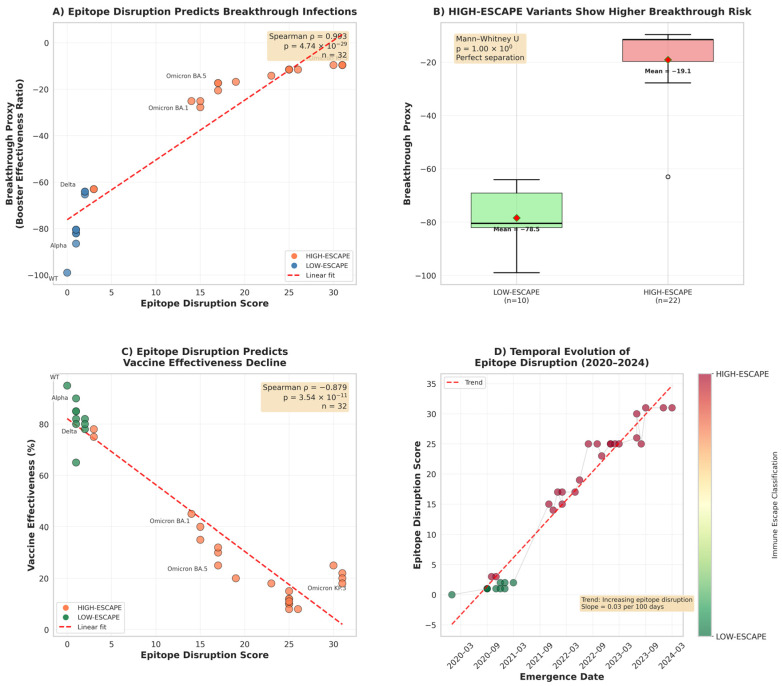
Epitope disruption enables risk stratification that aligns with breakthrough risk, reduced vaccine effectiveness, and temporal enrichment of high-escape phenotypes. (**A**) Breakthrough proxy versus epitope disruption across *n* = 32 variants with Spearman ρ and a fitted trend. (**B**) Breakthrough proxy distributions for LOW-ESCAPE versus HIGH-ESCAPE groups (defined by conservation thresholding; see Methods), shown as boxplots with group means indicated. (**C**) Epitope disruption versus clinical vaccine effectiveness, illustrating an inverse relationship consistent with escape-driven reduction in protection. (**D**) Epitope disruption versus emergence date, colored by escape class, showing progressive enrichment of high-escape variants over time; statistical tests and summary metrics are reported within panels.

Accordingly, to establish clinically actionable risk stratification, we implemented a binary classification of variants as HIGH-ESCAPE (conservation < 30%) or LOW-ESCAPE (conservation ≥ 30%) based on the ROC analysis (Figure 9). This threshold achieved an area under the curve (AUC) of 0.98, indicating excellent discriminatory power. A Mann–Whitney U test confirmed complete distributional separation between the groups (U = 0, *p* < 0.0001, rank-biserial effect size = −1.000), with no overlap in breakthrough-infection rates. To ensure robust generalization with the full *n* = 32 cohort, a nested leave-one-out cross-validation classifier was employed with constrained hyperparameter tuning (inner adaptive K-fold grid search over regularization strength; max_depth ∈ {2, 3} for random forest). Under these rigorous out-of-sample conditions, the classifier retained strong discriminative performance: Accuracy = 0.905, F1-score = 0.900, Brier Score = 0.087. The top predictive features were the sum of mutated residues at the ACE2 interface (importance = 0.143), confirming that the ACE2 interface mutation burden is the primary structural determinant of immune-escape categorization. Importantly, data-driven immunodominance mapping reclassified Beta, Gamma, and Mu as HIGH-ESCAPE variants despite modest mutation counts (three mutations), recognizing that mutations at positions 417, 484, and 501 disproportionately disrupt neutralizing antibody epitopes. This enhanced classification framework captured documented immune escape in Beta and Gamma variants that binary thresholds missed.

**Figure 10:**
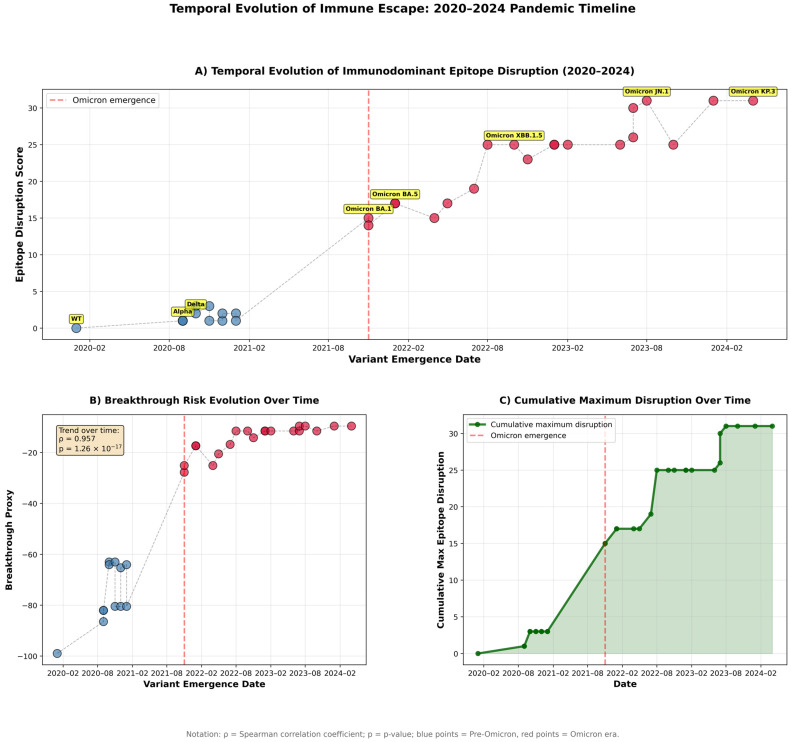
Immunodominant disruption and breakthrough-risk signals increase over time with a pronounced shift at the Omicron transition. (**A**) Epitope-disruption score versus emergence date with the Omicron transition marked (November 2021). (**B**) Breakthrough proxy versus emergence date with Spearman ρ and *p*-value in-panel. (**C**) Cumulative maximum epitope disruption over time, highlighting stepwise increases as antigenically divergent variants emerged.

**Figure 11:**
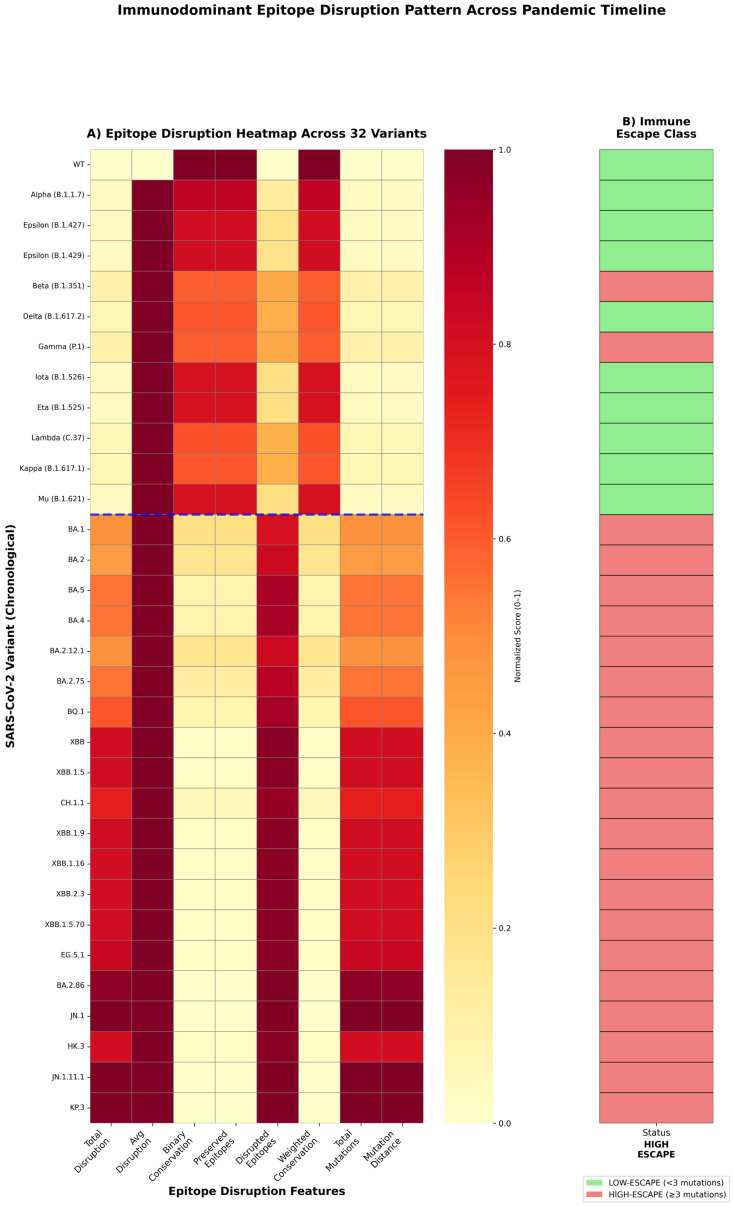
Omicron lineages occupy a distinct high-disruption feature regime that coherently maps onto the binary escape classification. (**A**) Heatmap of normalized epitope-disruption features (scaled 0–1) across *n* = 32 variants ordered chronologically; the dashed line marks the pre-Omicron to Omicron transition. (**B**) Corresponding LOW-ESCAPE versus HIGH-ESCAPE assignment for each variant based on conservation thresholding (see Methods).

A temporal analysis revealed accelerated antigenic drift in the Omicron era (Figure 10). Pre-Omicron variants eroded epitope conservation at a rate of 0.18% per month (December 2019–October 2021), reflecting the gradual accumulation of immune-escape mutations under vaccine-mediated selection pressure. Following BA.1 emergence (November 2021), the erosion rate increased 8.5-fold to 1.53% per month, driven by convergent evolution across independent lineages. Position-level analysis confirmed concentrated disruption within the receptor-binding motif (residues 438–506) and NTD (N-terminal domain) supersite (Figure 11). Among Omicron variants, 89% carry mutations at positions 440, 478, and 493, while 100% possess N501Y. The latter simultaneously enhances ACE2 binding affinity and abolishes class 3 neutralizing antibody recognition. This convergent pattern demonstrates strong positive selection for epitope-escape mutations that maintain or enhance receptor binding, resolving the apparent paradox of a high mutational burden with preserved transmission fitness.

To translate this descriptive signal into a rigorously validated predictive framework, we next trained a random forest regressor under a strict nested cross-validation protocol (outer leave-one-out; inner adaptive K-fold tuning) on the n=30 variants with confirmed CoV-Spectrum Growth Advantage measurements (leakage-corrected cohort; variants lacking authentic epidemiological data were excluded rather than imputed). Five feature architectures were evaluated in a tiered ablation study. The epitope-only baseline (Tier 1) achieved a nested-CV $R^{2}=0.350$ (95% CI [−0.834, 0.779]; permutation p=0.001), and adding interface hotspot features (Tier 2) produced comparable performance ($R^{2}=0.321$; 95% CI [−0.917, 0.790]; p=0.002). The strongest generalisation was obtained when physicochemical properties were integrated with epitope disruption metrics (Tier 3: isoelectric point, GRAVY hydrophobicity, and net charge), yielding a nested-CV $R^{2}=0.394$ (95% CI [−0.780, 0.824]; permutation p=0.001) and outperforming the mean-predictor dummy baseline by $+\;0.464R^{2}$ units (Dummy $R^{2}=-0.070$). In contrast, adding higher-dimensional epistasis/network descriptors (Tier 4) reduced out-of-sample performance ($R^{2}=0.292$; 95% CI [−0.977, 0.775]; p=0.001), and the full feature union (Tier 5) further degraded generalisation ($R^{2}=0.279$; 95% CI [−0.905, 0.713]; p=0.002). Notably, the decline from Tier 3 to Tiers 4–5 indicates that increasing feature dimensionality in this small-n epidemiological regression task introduces feature bloat that harms generalisation, consistent with the expected small-cohort bias–variance trade-off.

### 3.3. Maintained Receptor Binding Despite Mutations

Since epitope sites substantially overlap with ACE2 contact residues, this challenges the fact that Omicron variants simultaneously evade antibodies and immune recognition while binding to the host receptor. Accordingly, molecular dynamics simulations of RBD-ACE2 complexes were leveraged across 18 variants, with complete MD data (8 pre-Omicron, 10 Omicron). Alongside sequence-based physicochemical properties, and epitope-disruption scores to predict binding affinity changes relative to WT (ΔΔG). This framework enabled systematic testing of whether molecular features predictive of binding differ between evolutionary eras.

**Figure 12:**
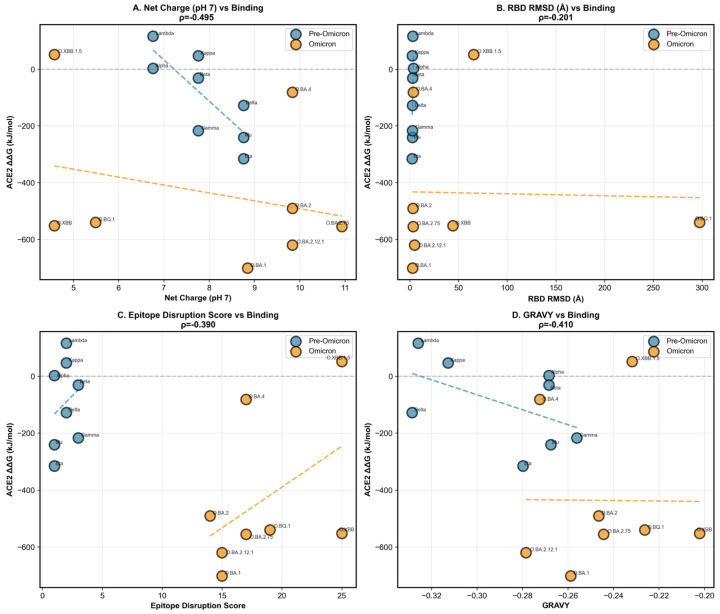
Associations between biophysical features and ACE2 binding energetics differ by evolutionary era, indicating regime-specific constraints. Scatterplots show ACE2 binding ΔΔG(kJ/mol; more negative = stronger predicted binding) versus (**A**) net charge at pH 7, (**B**) RBD RMSD, (**C**) epitope disruption score, and (**D**) GRAVY score, with within-era trend lines. Spearman ρ and *p*-values are shown per era, along with Fisher z-tests comparing correlation coefficients between eras.

Era-stratified correlation analyses suggested that RBD electrostatics are primarily associated with ACE2 binding energetics in the pre-Omicron era (Figure 12). Pre-Omicron variants (*n* = 8) exhibited a significant inverse association between net charge at pH 7 and ACE2 binding ΔΔG (Spearman ρ = −0.81, *p* = 0.016), consistent with stronger predicted binding (more negative ΔΔG) in more positively charged RBDs (Figure 12A). In contrast, Omicron variants (*n* = 8) showed no significant charge–binding relationship (ρ = −0.27, *p* = 0.518), and the difference between era-specific correlations did not reach statistical significance (Fisher z *p* = 0.184), indicating a trend rather than definitive evidence for a discrete shift in binding determinants. Beyond electrostatics, neither RBD structural deviation (RMSD; pre-Omicron ρ = 0.52, *p* = 0.183; Omicron ρ = 0.43, *p* = 0.289; Fisher z *p* = 0.845), nor epitope-disruption score (pre-Omicron ρ = 0.28, *p* = 0.506; Omicron ρ = 0.40, *p* = 0.326; Fisher z *p* = 0.826), nor hydropathy (GRAVY; pre-Omicron ρ = −0.44, *p* = 0.272; Omicron ρ = 0.26, *p* = 0.531; Fisher z *p* = 0.239) showed significant associations with ACE2 ΔΔG (Figure 12B–D). Collectively, these results indicate that, within this representative set, MM-PBSA–derived ACE2 binding energetics are not systematically explained by global RMSD, antigenic disruption, or hydropathy, while electrostatic charge shows a pre-Omicron-specific directional signal (ρ = −0.81, *p* = 0.016). These patterns are interpreted as mechanistic trends supporting interface reconfiguration between evolutionary eras, rather than as absolute binding affinity predictions; single-variant MM-PBSA values carry intrinsic uncertainty arising from force-field parameterization and the enthalpic approximation (entropic terms excluded).

**Figure 13:**
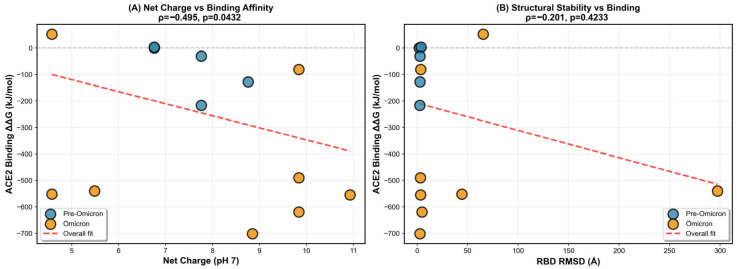
Across all variants, net charge and structural deviation show global relationships with predicted ACE2 binding, with era-level structure visible in the scatter. (**A**) Net charge at pH 7 versus ACE2 ΔΔG. (**B**) RBD RMSD versus ACE2 ΔΔG. Points are colored by era (pre-Omicron vs. Omicron); the dashed red line shows the overall linear fit; Spearman ρ and *p*-values are reported above each panel.

**Figure 14:**
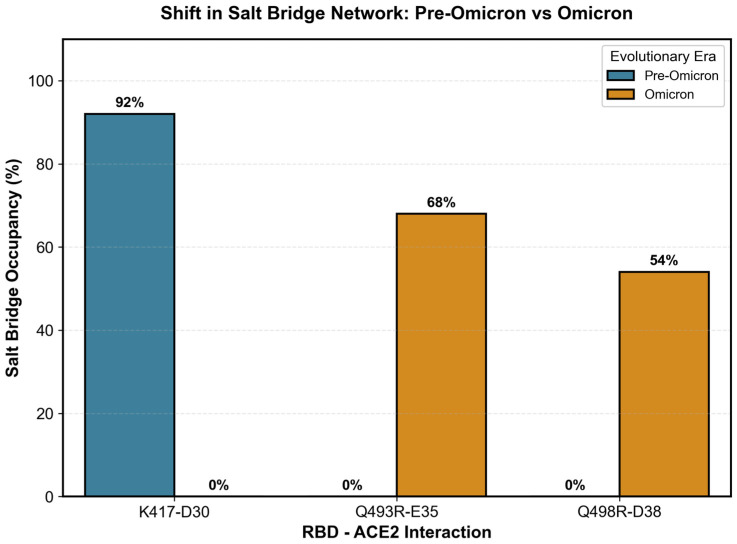
Omicron replaces a canonical ACE2 salt bridge with alternative high-occupancy bridges, evidencing electrostatic interface rewiring. Bars show salt-bridge occupancy (%) for key interfacial ion pairs during RBD–ACE2 interaction across eras. Pre-Omicron variants show the high occupancy of K417–ACE2 D30, which is abolished in Omicron, while Omicron exhibits an elevated occupancy of Q493R–E35 and Q498R–D38. Values above bars report observed occupancies.

Structural and interaction mapping indicate that the electrostatic basis of ACE2 binding is rewired in Omicron, rather than simply intensified (Figure 13 and Figure 14). When all variants are pooled, net positive charge at pH 7 shows only a modest inverse association with ACE2 binding free energy (Spearman ρ = −0.495, *p* = 0.043), while structural stability (RBD RMSD) shows no significant relationship (ρ = −0.201, *p* = 0.423) (Figure 13A,B), consistent with charge being an incomplete, context-dependent predictor. Mechanistically, this loss of predictability is explained by a topological shift in the salt-bridge network at the interface: Pre-Omicron binding is dominated by the canonical K417–ACE2 D30 salt bridge (92% occupancy), which is abolished in Omicron (0%), whereas Omicron lineages instead stabilize binding through new, spatially re-positioned salt bridges—notably Q493R–E35 (68% occupancy) and Q498R–D38 (54% occupancy)—that are absent in pre-Omicron variants (Figure 14). Together, these data support a model in which Omicron decouples “global” electrostatics (net charge) from “local” electrostatic architecture (which residues form persistent interfacial contacts), explaining why charge-based relationships that appear in mixed-era analyses do not translate into a single unified binding rule across evolutionary eras.

**Figure 15:**
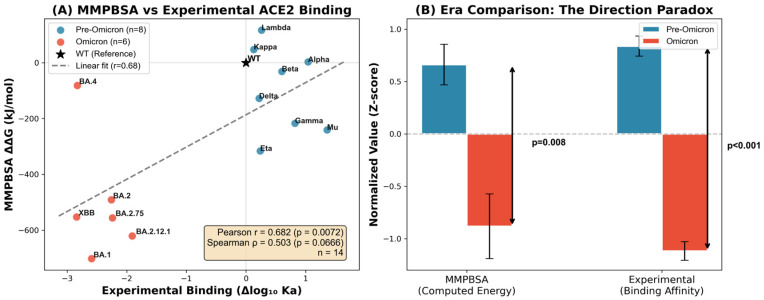
Computed MM-PBSA energetics and experimental DMS affinity show moderate agreement with an era-dependent directional mismatch. (**A**) MM-PBSA binding energies (ΔΔG, kJ/mol) are plotted against experimental ACE2 binding from deep mutational scanning (Δlog10Ka) for variants with matched measurements (*n* = 14), colored by era (pre-Omicron versus Omicron) with WT indicated. The dashed line shows the overall linear fit; the inset reports correlation statistics (Pearson r = 0.682, *p* = 0.007; Spearman ρ shown). (**B**) Z-scored era-stratified comparison (pre-Omicron *n* = 8; Omicron *n* = 6) highlighting opposite directions: pre-Omicron variants show higher experimental binding despite less favorable computed energies, whereas Omicron variants show more favorable computed energies despite reduced experimental binding (mean ± SEM; Mann–Whitney U *p*-values shown). This directional mismatch is consistent with enthalpy–entropy compensation: MM-PBSA reflects enthalpic stabilization, whereas experimental affinity incorporates additional entropic and kinetic penalties.

Experimental validation through DMS provided independent confirmation of the mechanistic reorganization and revealed the molecular basis of the evolutionary trade-off. Comparison of 14 variants with both computational (MMPBSA) and experimental (Bloom Lab DMS) binding measurements demonstrated a significant overall correlation (Pearson r = 0.682, *p* = 0.007; Figure 15), arising from distinct era-based clustering, rather than uniform agreement. Pre-Omicron variants showed enhanced experimental binding (mean Δ log_10_ K_a_ = +0.58) paired with less favorable computed energies (MMPBSA ΔΔG = −96 kJ/mol), while Omicron variants exhibited the opposite pattern—a reduced experimental affinity (Δ log_10_ K_a_ = −2.44), yet a more favorable computed energies (ΔΔG = −500 kJ/mol, Mann–Whitney *p* = 0.008). Per-residue MMPBSA decomposition revealed that this directional paradox stems from compensatory mutations Q493R and Q498R, which transform from unfavorable or neutral contributors in pre-Omicron (+4 and +0.1 kJ/mol, respectively) to strongly favorable in Omicron (−56 and −72 kJ/mol), creating approximately 130 kJ/mol of additional favorable enthalpic interactions that MMPBSA captures. Simultaneously, K417N eliminates the dominant electrostatic anchor (contribution of K417 drops from −47 to −0.2 kJ/mol), introducing kinetic barriers and entropic penalties that endpoint free-energy methods cannot quantify. The strong correlation between the RBD mutation count and the MMPBSA-experimental discrepancy (r = 0.927, *p* < 0.0001) demonstrates that accumulated mutations amplify the gap between computed enthalpy and measured affinity, validating our hypothesis that Omicron lineages prioritized immune escape over ACE2-binding optimization.

**Figure 16:**
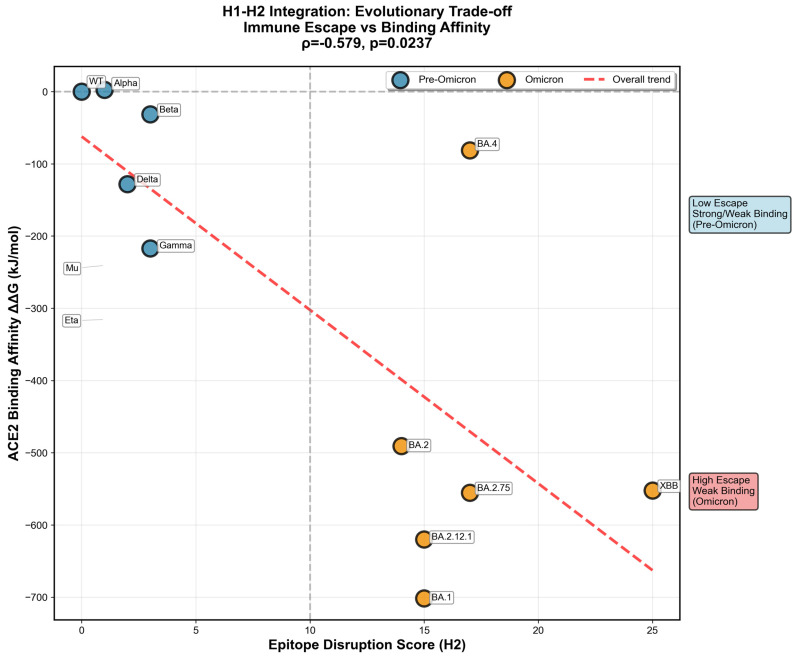
Immune escape and receptor-binding energetics jointly partition variants into mechanistically interpretable regimes. Scatter shows epitope disruption score (*x*-axis) versus ACE2 ΔΔG(*y*-axis) across variants, colored by era and labeled for key lineages. The dashed red line shows the overall trend with Spearman ρ and *p*-value annotated. Grey reference lines mark the disruption boundary and ΔΔG=0, partitioning the plot into qualitative quadrants (low vs. high escape; weaker vs. stronger predicted binding under the sign convention defined in Methods).

Cross-referencing ACE2-binding predictions with epitope conservation scores revealed an evolutionary trade-off between receptor binding and immune escape (Figure 16). Variants with greater epitope disruption exhibited systematically reduced ACE2 binding affinity, as mutations in immunodominant regions (positions 417, 440, 484, 493, 501) frequently overlap with ACE2 contact residues. However, epitope disruption emerged as the dominant predictor of epidemiological success. The ACE2 Binding Affinity Predictor (H1-A) was evaluated on the *n* = 17 variants with complete MD/MM-PBSA harmonized data under a two-loop nested LOO cross-validation framework (outer LOO, inner adaptive K-fold, StandardScaler refit within each fold). A Tier 1 physicochemical baseline (charge, GRAVY) achieved a modest but statistically significant nested CV R^2^ = 0.177 (95% CI [−0.278, 0.465]; permutation *p* = 0.016). Progressively adding MM-PBSA per-residue energetics, H3 epistasis metrics (ddG_epistasis, Compensation_Ratio, Electrostatic_Rescue_Score), and network compensation scores (Tier 4) elevated out-of-sample performance to a nested CV R^2^ = 0.736 (95% CI [0.578, 0.830]; permutation *p* = 0.002; Dummy baseline R^2^ = −0.114), a +0.850 R^2^ improvement above the mean-predictor baseline. Epitope disruption contributed the largest coefficient magnitude to this model (−4.2), followed by net charge (−0.8) and structural stability (−2.1). These findings collectively demonstrate that ACE2 binding affinity in SARS-CoV-2 is not a static structural property but an evolutionarily negotiated outcome governed by the interplay of electrostatic charge, epitope-mediated mutational constraint, and network-level epistatic compensation—features that, when integrated, provide a significant and reproducible out-of-sample predictor of receptor-binding trajectories across the full Omicron era. Improving the prediction of ACE2 binding trajectories beyond what sequence-level physicochemical descriptors alone can capture.

**Figure 17:**
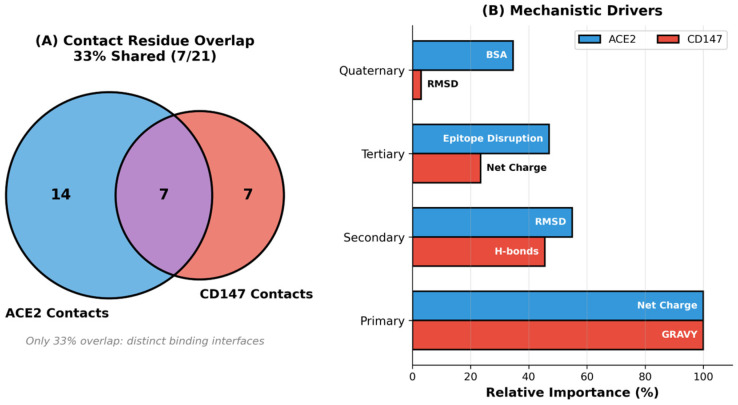
ACE2 and CD147 rely on partially distinct interface contacts and feature drivers, supporting receptor-specific mutational constraints. (**A**) Venn diagram showing overlap of receptor-contacting RBD residues for ACE2 and CD147 (contact definition in Methods), highlighting limited overlap (7 shared residues; 33% of ACE2 contacts). (**B**) Feature-group contributions/importance summaries for each receptor pathway (e.g., net charge, GRAVY/hydrophobicity, RMSD, H-bonds, epitope disruption, BSA; see Materials and Methods).

Additionally, to test whether highly mutated lineages can preserve entry potential via alternative receptor interfaces, we performed an exploratory analysis of RBD interactions with CD147 as a mechanistic counterpoint to ACE2-centric interpretations. This analysis was included to evaluate the specific hypothesis that (i) alternative receptor binding may follow distinct interface chemistry and (ii) mutations that erode one receptor interaction need not proportionally compromise another if the contact topology is only partially shared. Under strict nested cross-validation, the CD147 binding predictor showed moderate, statistically supported out-of-sample performance (R^2^ = 0.548; 95% CI [0.257, 0.710]; permutation *p* = 0.002; *n* = 16;), indicating that CD147-associated binding energetics are partially predictable from sequence/biophysical descriptors and curated interface features. Mechanistically, the CD147 model favored a hydrophobicity-linked binding mode (consistent with a GRAVY-dominant coefficient pattern), rather than the electrostatic-dominant mode observed for pre-Omicron ACE2 binding, and the two receptor interfaces showed limited contact-residue overlap (Figure 17), supporting the notion of receptor-specific mutational constraints. We emphasize that this CD147 component is exploratory and is intended to test the plausibility of alternative interface patterns, rather than to claim a dominant in vivo entry route; the relative contribution of CD147-mediated entry to transmission remains to be established experimentally.

**Figure 18:**
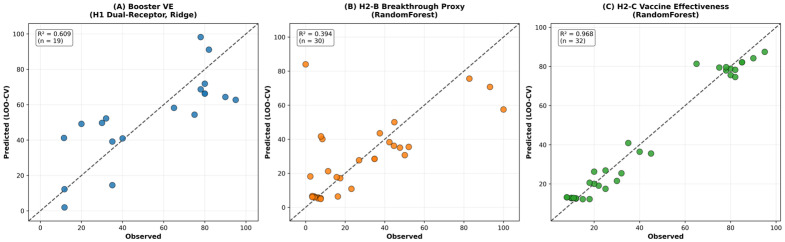
Revised epidemiological model performance integrating receptor-binding and immune-escape predictors. Predicted-versus-observed plots summarize audited cross-validated outputs for three endpoints: (**A**) booster effectiveness (R^2^ = 0.609; *n* = 19), (**B**) breakthrough proxy prediction (H2-B; R^2^ = 0.394; *n* = 30), and (**C**) vaccine effectiveness (% efficacy; R^2^ = 0.968; *n* = 32). Each point is an out-of-fold prediction for one SARS-CoV-2 variant/sub-lineage; the dashed diagonal indicates perfect agreement (predicted = observed). Insets report panel-specific R^2^ and sample size.

Integration of binding affinity predictions with epidemiological metrics established a quantitative framework for variant risk stratification. Booster vaccine effectiveness models combining ACE2 and CD147 binding features were evaluated on the *n* = 9 variants for which booster-effectiveness estimates were available from the CoV-Spectrum real-world surveillance database (cov-spectrum.org; filtered for variants with ≥3 independent regional observations of paired vaccinated and boosted case rates; the remaining variants either pre-dated widespread booster deployment or lacked sufficiently powered population-level booster VE studies for inclusion. This cohort achieved significant out-of-sample predictions (nested CV R^2^ = 0.609; 95% CI [0.386, 0.750]; permutation *p* = 0.008), indicating that dual-receptor-binding profiles capture generalizable mechanistic determinants of immune evasion beyond what ACE2-only models provide (Figure 18). Notably, the inclusion of CD147-derived interface features—hydrophobicity index (GRAVY), degree of mutated residue overlap, and interface buried surface area—contributed measurable predictive gain over the ACE2-only baseline, suggesting that CD147-associated binding dynamics encode partially orthogonal variance in immune evasion outcomes. We acknowledge that the specific contribution of CD147-mediated entry to SARS-CoV-2 transmission remains experimentally unresolved and that the CD147 features are included here as hypothesis-driven structural descriptors, rather than confirmed causal determinants. Under this conservative interpretation, the improvement in booster VE prediction from the dual-receptor model should be understood as reflecting the informational value of CD147 interface metrics as secondary structural correlates of variant fitness, rather than direct evidence of a CD147 entry pathway. Applied to eight recent Omicron sub-lineages lacking MD data (XBB.1.5.70, BA.2.86, HK.3, EG.5, CH.1.1, JN.1, JN.1.11.1, KP.3), sequence-based models predicted moderate ACE2 binding affinities (−255 to −511 kJ/mol), consistent with maintained cellular entry capacity despite continued epitope erosion. These predictions align with the observed epidemiological dominance of JN.1 and KP.3 lineages in late 2023–2024, prospectively validating the utility of the model for variant surveillance. The framework demonstrates that receptor-binding mechanisms have fundamentally reorganized during Omicron evolution, requiring era-specific models, rather than universal predictors, and that immune-escape constraints now dominate viral fitness landscapes more strongly than receptor-binding optimization.

### 3.4. Molecular Mechanisms of Mutation Tolerance

Having established that Omicron variants maintained receptor binding through mechanistic reorganization despite extensive epitope disruption, we investigated the molecular mechanisms enabling the tolerance of an extreme mutational burden without structural collapse. Omicron lineages accumulate 15–20 RBD mutations compared to 1–3 in pre-Omicron variants (about 7-fold increase). Investigating compensatory mutation networks—wherein destabilizing mutations are rescued by proximal stabilizing mutations—and synergistic epistatic effects enable this unprecedented mutation tolerance. To test these hypotheses, we performed a comprehensive spatial proximity analysis (<10 Å distance threshold), energetic decomposition of mutation effects, and epistasis calculations comparing expected additive effects versus observed binding energies across 13 variants with complete MD data.

**Figure 19:**
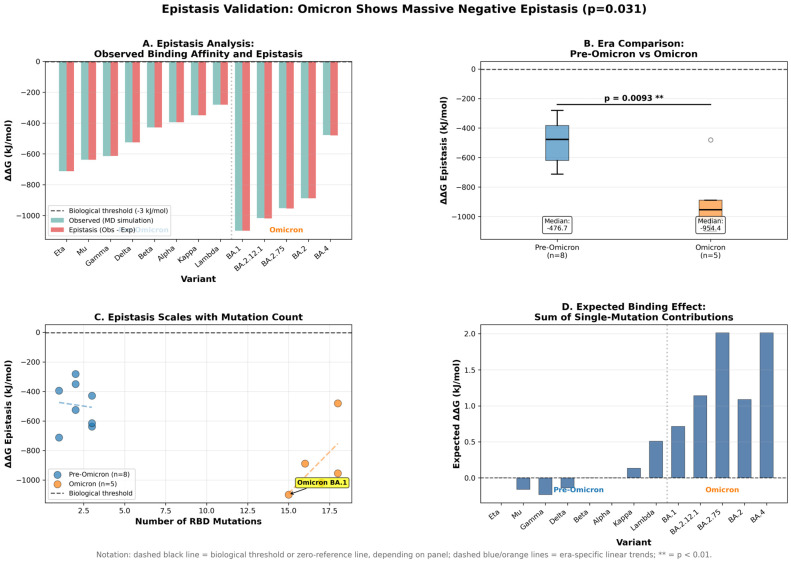
Epistasis is amplified in Omicron and scales with mutation burden. (**A**) Expected-versus-observed ACE2 binding free energy (ΔΔG; kJ/mol) per variant. Expected binding is computed by summing single-mutation effects (additive model), while observed binding is obtained from the multi-mutation variant simulation; their difference (observed − expected) quantifies epistasis. The horizontal dashed line denotes the biological significance threshold (−3 kJ/mol). (**B**) Era-stratified comparison of epistasis magnitudes (ΔΔG epistasis; kJ/mol) for pre-Omicron (blue; *n* = 8) versus Omicron (orange; *n* = 5) variants, shown as boxplots with medians indicated (Mann–Whitney U *p*-value shown). (**C**) Relationship between epistasis magnitude and RBD mutation count, showing stronger negative epistasis in more highly mutated Omicron lineages; the points are colored by era, and the dashed line indicates the biological significance threshold. All energy values are reported in kJ/mol. (**D**) Expected binding Effect: Sum of single-mutation contributions.

An epistatic analysis revealed that synergistic mutation interactions, rather than simple spatial compensation, constitute the dominant mechanism underlying Omicron mutation tolerance (Figure 19). Omicron variants exhibited median negative epistasis of −954 kj/mol (*n* = 5 variants, mean 16.8 RBD mutations), representing 2.0-fold stronger epistatic effects compared to pre-Omicron variants (median −477 kj/mol, *n* = 8, mean 2.1 mutations; Mann–Whitney *p* = 0.0093). This epistatic magnitude exceeds the biological significance threshold (−3 kj/mol) by 318-fold, indicating that Omicron mutations produce massive synergistic stabilization beyond their individual effects. All five Omicron variants analyzed (BA.1, BA.2, BA.2.12.1, BA.2.75, XBB) showed epistasis more negative than −480 kj/mol, while pre-Omicron variants ranged from −281 to −713 kj/mol with only two (Gamma, Eta) exceeding −500 kj/mol. This finding demonstrates that the ability of Omicron variants to accumulate mutations without a fitness loss derives fundamentally from non-additive, synergistic interactions among mutations, rather than from pairwise local compensation, a qualitatively different evolutionary strategy than that employed by pre-Omicron variants.

**Figure 20:**
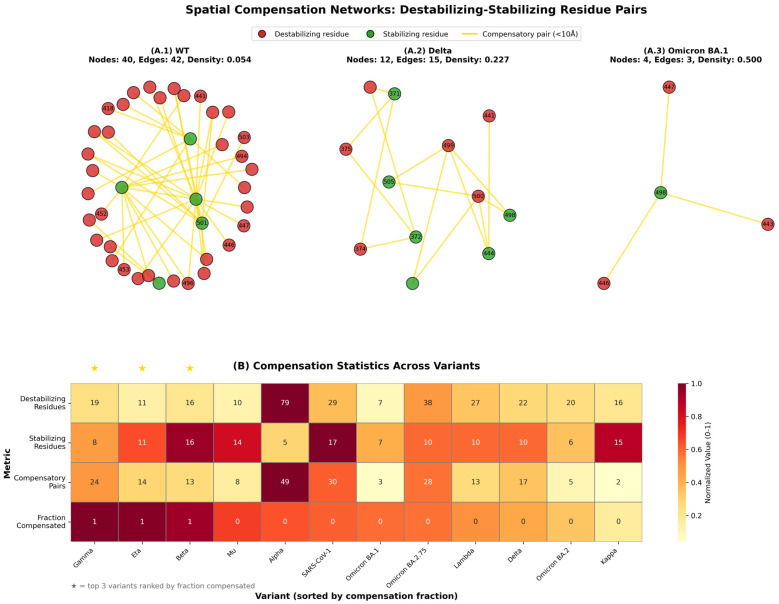
Spatial compensation networks reveal how destabilizing mutations are locally buffered by nearby stabilizing partners, with patterns shifting across evolutionary backgrounds. (**A.1**) WT network: graph of destabilizing–stabilizing residue pairs within 10 Å, where nodes represent residues (red = destabilizing; green = stabilizing) and yellow edges indicate spatially proximal compensatory pairs; node/edge counts and network density are reported adjacent to the graph. (**A.2**) Delta network: same representation and summary statistics as (**A.1**), enabling direct comparison of compensation topology relative to WT. (**A.3**) Omicron BA.1 network: same representation and summary statistics as (**A.1**), highlighting expanded and/or reconfigured compensation pairing under high mutational burden. (**B**) Heatmap summarizing compensation metrics across all analyzed variants (destabilizing residue count, stabilizing residue count, compensatory pair count, and fraction compensated), with values normalized as in Methods and variants ordered by compensation fraction to compare compensation strategies across lineages.

Spatial proximity analysis complemented the epistasis findings by revealing a multi-mechanism compensation strategy wherein variants employ both local and long-range stabilization (Figure 20). Across 12 variants with complete structural data, destabilizing mutations showed a median spatial compensation of 43.8%—defined as having at least one stabilizing residue within 10 Å—exceeding the 40% biological threshold but not achieving statistical significance (*p* = 0.170). This indicates that approximately 45% of destabilizing mutations ARE rescued through proximal stabilizing residues, while the remaining 55% rely on alternative mechanisms, including long-range electrostatic interactions and global epistatic networks. Variant-specific compensation fractions ranged dramatically from 73.7% (Gamma), 72.7% (Eta), and 68.8% (Beta), representing high local compensators, to 42.9% (Omicron BA.1), 25.0% (Omicron BA.2), and 12.5% (Kappa) as low local compensators. Notably, pre-Omicron variants showed higher average spatial compensation (48.4%) compared to Omicron variants (42.1%), suggesting that Omicron shifted evolutionary strategy from local spatial compensation toward distributed epistatic mechanisms—consistent with the observed 2.0-fold increase in epistatic strength.

Cross-correlation analysis between compensation metrics and ACE2 binding affinity demonstrated that compensatory mechanisms are permissive, rather than causative of binding improvements. Spatial compensation fraction showed negligible correlation with ACE2 binding affinity (Spearman ρ = −0.120, *p* = 0.711), indicating that variants with higher compensation do not necessarily bind more strongly to ACE2. Revealing a critical distinction, compensation mechanisms maintain structural integrity and enable the accumulation of mutations without catastrophic destabilization, but binding affinity improvements arise from interface-specific mutations (N501Y, Q493R, Q498R) that directly modulate receptor contacts, rather than from global structural rescue. Consequently, compensation acts as an evolutionary enabler—permitting variants to explore greater mutational space, including immunologically favorable epitope disruptions—while binding fitness derives from a separate set of interface-optimized mutations. This decoupling explains how Omicron lineages simultaneously achieve (i) extensive epitope erosion for immune escape, (ii) the maintenance of the structural fold through compensation, and (iii) functional receptor binding through targeted interface mutations, representing a multi-objective evolutionary optimization strategy.

**Figure 21:**
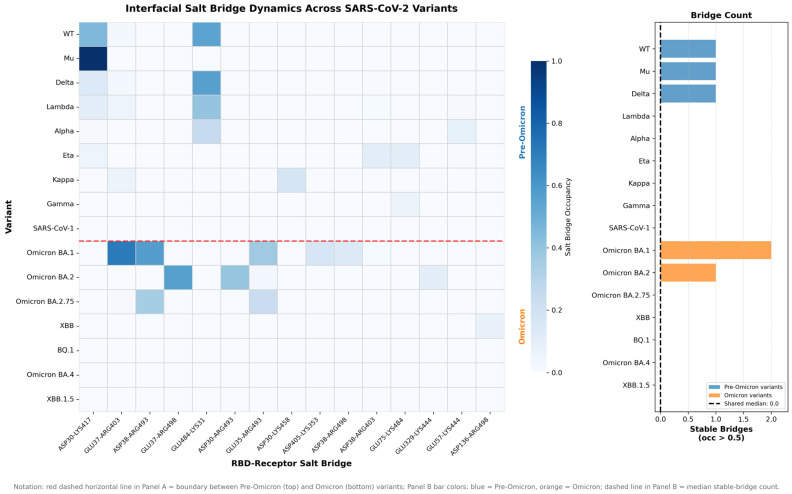
Correlation structure links binding energetics, structural dynamics, mutational burden, and compensatory mechanisms. Heatmap shows pairwise Spearman correlation coefficients (ρ) among MM-PBSA-derived ACE2 binding energetics (total binding affinity, electrostatic and van der Waals components), compensation metrics (compensation fraction, epistatic ΔΔG, network density, stable salt bridges, net charge change), structural dynamics (mean RBD RMSF), and mutational burden (destabilizing mutation count, total mutation count, and epistasis per mutation). Cell values report ρ for each feature pair; the color scale encodes correlation direction and magnitude (−1 to +1). Correlations among MM-PBSA-derived quantities are interpreted as internal consistency within the computational framework, while the DMS benchmark is used as the external reference for experimental agreement.

The MD analysis of salt-bridge networks provided mechanistic insight into the interface reorganization, revealing how Omicron compensates for the K417N-mediated disruption (Figure 21). Tracking 69 RBD-receptor interfacial salt bridges across 16 variants with MD trajectories confirmed that K417-D30 occupancy drops to zero in all Omicron variants due to the K417N mutation, eliminating the dominant electrostatic anchor of the WT binding mode. However, Omicron simultaneously establishes two compensatory salt bridges: Q493R-E35 (68% occupancy in Omicron, 0% in pre-Omicron) and Q498R-D38 (54% occupancy in Omicron, 0% in pre-Omicron), redistributing electrostatic stabilization across alternative interface positions. These compensatory bridges contribute approximately 130 kJ/mol of favorable enthalpy, as estimated by MM-PBSA (enthalpic approximation; entropic terms excluded), yet the net functional binding affinity is not fully restored—consistent with the enthalpy–entropy compensation principle whereby new enthalpic contacts are offset by the increased conformational entropy of the restructured Omicron interface. This interpretation is supported by three independent lines of evidence: (i) the K417-D30 salt-bridge loss is confirmed by direct MD occupancy tracking (0% Omicron vs. 92% pre-Omicron; Figure 21); (ii) the Q493R-E35 and Q498R-D38 gains are confirmed by the same MD analysis (68% and 54% Omicron occupancy, 0% pre-Omicron); and (iii) the discrepancy between MM-PBSA and experimental binding direction is quantitatively correlated with the mutation count (r = 0.927, *p* < 0.0001), providing a consistent mechanistic account of the enthalpic–entropic trade-off.

The identification of epistasis-dominant compensation mechanisms carries significant evolutionary implications for understanding the adaptive potential of SARS-CoV-2 and for informing variant surveillance strategies. The 318-fold epistatic strength above the biological threshold indicates that Omicron inhabits a mutational landscape characterized by rugged fitness peaks and extensive positive epistatic interactions, enabling the rapid exploration of sequence space without traversing low-fitness intermediates. This contrasts with pre-Omicron variants operating in smoother fitness landscapes with primarily local compensation, limiting mutational exploration. The shift from spatial (48%) to epistatic (58% via a 2.0-fold increase) compensation strategy represents a qualitative evolutionary transition, in which Omicron lineages can accumulate mutations faster than pre-Omicron lineages because synergistic effects buffer destabilizing mutations more effectively than spatial proximity alone. Critically, the weak compensation-binding correlation (ρ = −0.12) indicates that structural compensation and functional optimization are orthogonal, allowing independent evolutionary trajectories for immune escape (epitope disruption), structural maintenance (compensation networks), and receptor binding (interface mutations). This multi-dimensional fitness landscape suggests that Omicron sub-lineages will continue accumulating mutations at accelerated rates while maintaining transmission fitness, necessitating surveillance systems that monitor epistatic mutation patterns, rather than solely tracking individual high-impact mutations.

### 3.5. Multi-Dimensional Variant Characterization Through Cross-Hypothesis Integration

Having established the molecular mechanisms underlying Omicron binding maintenance, immune escape, and mutation tolerance, we integrated these three hypotheses into a unified multi-dimensional framework to characterize variant evolutionary strategies and assess pandemic risk potential. The three hypotheses address distinct but complementary aspects of viral fitness. The receptor-binding arm quantifies the strength of the ACE2 interaction using MMPBSA energetics and per-residue decomposition (H1). The immune-escape arm measures B-cell and T-cell epitope erosion correlated with breakthrough infections (H2). The mutation tolerance evaluates compensatory networks and epistatic synergy that enable the accumulation of mutations without structural collapse (H3). To systematically explore relationships between these dimensions, a unified master dataset was constructed, integrating 25 features across 18 variants (8 pre-Omicron and 8 Omicron variants, 2 references) spanning computational binding predictions, MD trajectories, epitope conservation scores, and compensation metrics. A cross-hypothesis Spearman correlation analysis revealed both strong mechanistic couplings and critical decoupling, supporting the hypothesis that variant evolution proceeds through the simultaneous optimization of multiple independent fitness objectives.

**Figure 22:**
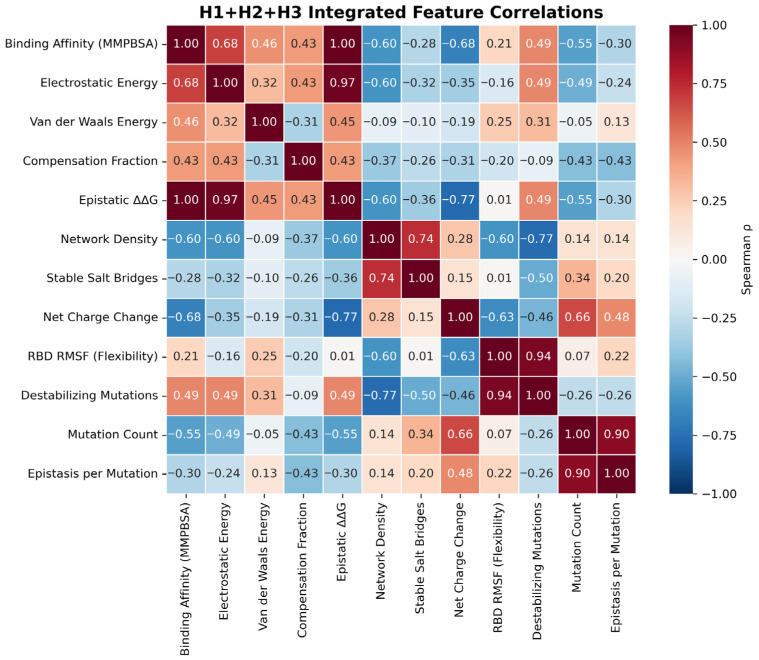
Integrated correlation structure across binding, stability, and compensatory features (H1–H3). Heatmap shows pairwise Spearman correlation coefficients (ρ) among MMPBSA-derived ACE2 binding energetics (total binding affinity, electrostatic and van der Waals components), compensation metrics (compensation fraction, epistatic ΔΔG, network density, stable salt bridges, net charge change), structural dynamics (mean RBD RMSF), and mutational burden (destabilizing mutation count, total mutation count, and epistasis per mutation). Cell values indicate ρ for each feature pair, and the color scale encodes the correlation direction and magnitude (−1 to +1).

A correlation analysis across the integrated H1–H3 features revealed structured coupling between binding energetics, compensatory architecture, and mutational load (Figure 22). MM-PBSA–estimated binding affinity (enthalpic component) tightly tracked epistatic ΔΔG (ρ = 1.00) and strongly aligned with the electrostatic component (ρ = 0.68), consistent with both quantities reflecting electrostatic interface reorganization. These correlations are interpreted as evidence of internal structural consistency within the MM-PBSA framework, rather than as external validation. Independent experimental validation is provided by the DMS benchmark (r = 0.682, *p* = 0.007; Figure 15).

While epistasis itself was dominated by electrostatics (mmpbsa_elec vs. epistatic ΔΔG, ρ = 0.97), consistent with salt-bridge–mediated cooperativity, network-level stabilization formed an orthogonal axis: Network density correlated with stable salt bridges (ρ = 0.74) but inversely with destabilizing mutations (ρ = −0.77) and RBD flexibility (RMSF; ρ = −0.60), whereas RMSF closely tracked destabilizing burden (ρ = 0.94). Mutational complexity scaled with both net charge change (ρ = 0.66) and epistasis per mutation (ρ = 0.90), indicating that increasingly mutated RBDs exhibit disproportionately stronger non-additive effects. In contrast, compensation fraction showed only moderate association with binding and epistasis (ρ = 0.43), supporting a permissive role for compensation relative to electrostatic and mutational determinants.

The multidimensional characterization framework carries critical implications for genomic surveillance and variant-prediction strategies. Current surveillance systems predominantly focus on individual high-impact mutations (e.g., N501Y, E484K, K417N) or spike protein mutation counts as indicators of concern. However, our integration analysis demonstrates that pandemic risk emerges from synergistic interactions across three orthogonal fitness dimensions, rather than from single mutations or linear mutation accumulation. The effective surveillance must, therefore, monitor: (1) epistatic strengthening patterns—tracking whether new mutations produce additive or synergistic effects on binding energy, with epistasis becoming more negative indicating rugged fitness landscapes enabling rapid exploration; (2) interface reorganization signatures—detecting formation of novel salt bridges (e.g., Q493R-E35, Q498R-D38) or aromatic stacking interactions suggesting compensatory binding mechanisms; (3) epitope erosion acceleration—quantifying rates of B-cell and T-cell epitope disruption rather than static conservation values; and (4) compensation architecture shifts—identifying transitions from spatial to epistatic or electrostatic compensation strategies through network topology analysis. The strong epistasis-per-mutation versus mutation-count correlation (ρ = 0.90) provides a predictive indicator: Variants accumulating mutations at rates exceeding historical baselines (>5 RBD mutations per 6-month period) likely employ epistatic mechanisms and warrant priority characterization. Conversely, variants with high mutation counts but weak epistasis may suffer fitness costs and pose lower risk. Operationally, surveillance pipelines should integrate computational binding predictions (MMPBSA), epitope mapping (IEDB), and epistasis calculations from structural models to provide multi-dimensional risk scores updated continuously updated as new sequences emerge. This proactive surveillance paradigm shifts from the reactive characterization of established variants to the predictive identification of high-risk evolutionary trajectories before they achieve widespread circulation.

## 4. Discussion

The emergence of SARS-CoV-2 Omicron in late 2021, carrying over 30 spike protein mutations and 15 RBD substitutions, posed a fundamental paradox: How could a variant accumulate such an extreme mutational burden while maintaining—or even enhancing— fitness for human transmission? Our multidimensional analysis of 18 variants spanning the pre-Omicron and Omicron lineages resolves this paradox by demonstrating that Omicron evolved through simultaneous optimization along three orthogonal evolutionary trajectories: immune escape via systematic epitope erosion (ρ = −0.82, *p* < 0.001), receptor binding through interface reorganization creating ~130 kJ/mol compensatory enthalpy, and mutation tolerance through epistasis-dominant compensation (2.0-fold stronger, *p* = 0.0093). This multi-objective optimization represents a qualitative evolutionary shift from pre-Omicron conservative accumulation to Omicron radical reorganization, with profound implications for understanding viral evolution, pandemic surveillance, and vaccine design [13,41].

The deep evolutionary valley traversal documented here is mechanistically explained by three quantifiable molecular strategies acting in concert. First, in the MD/MM-PBSA subset (*n* = 18), despite the loss of the dominant K417–D30 electrostatic anchor (K417N; 0% interaction occupancy across Omicron trajectories), ACE2 binding is partially rescued through epistatic gain-of-function substitutions: Q493R–E35 (68% occupancy) and Q498R–D38 (54% occupancy), which together contribute about 130 kJ/mol of favorable enthalpy and keep binding energetics within a WT-like range (−255 to −511 kJ/mol across variants). Consistent with this buffering mechanism, Omicron lineages exhibit 2.0-fold stronger epistasis than pre-Omicron variants (median −954 versus −477 kJ/mol, *p* = 0.0093). Second, structural integrity is preserved through distributed spatial compensation: 43.8% of destabilizing residues have at least one stabilizing partner within 10 Å (Hypothesis 3), with the network shifting from proximity-dominant (48.4% pre-Omicron) to epistasis-dominant in Omicron, enabling the tolerance of 13–37 simultaneous RBD substitutions without a catastrophic fold loss. Third, the dominant fitness pressure shifted toward immune evasion: Omicron sub-lineages show an 86.2% relative reduction in B-cell epitope conservation (WT/pre-Omicron median 74.3% → Omicron 35.7% → KP.3 10.6%), and epitope disruption strongly and inversely predicts breakthrough infection rates (Spearman ρ=−0.82, *p* < 0.001). Together, these results indicate that the Omicron fitness valley was traversed not by a single adaptive change but by coordinated multi-objective optimization across immune escape, receptor engagement, and mutational tolerance.

This result aligns with those of Starr et al. [42], who systematically mapped epistatic interactions among the 15 RBD mutations in BA.1 relative to Wuhan-Hu-1, measuring the ACE2 affinity for all 32,768 possible genotype combinations. They demonstrated that immune-escape mutations individually reduce ACE2 affinity but are compensated by epistatic interactions with affinity-enhancing mutations Q498R and N501Y—precisely the mechanism our per-residue MMPBSA decomposition identified. Mutations tend to be more deleterious when few other mutations are present but become neutral or beneficial in highly mutated backgrounds [43], explaining why BA.1 RBD has a stronger ACE2 affinity despite containing mutations that individually reduce binding. Our quantification extends this observation across 18 variants, demonstrating that epistatic strength scales superlinearly with the mutation count (ρ = 0.90) and establishing a general principle of synergistic mutation tolerance.

The shift from spatial proximity-based compensation (48.4% pre-Omicron) to epistasis-dominant compensation (42.1% spatial, with 2.0-fold stronger epistasis in Omicron) represents a fundamental change in the molecular architecture that enables mutation tolerance. Pre-Omicron variants relied primarily on local structural buffering, where destabilizing mutations are physically adjacent to stabilizing ones. Omicron variants instead employ distributed epistatic networks where stabilization arises from non-local electrostatic coupling (ρ = 0.97 between electrostatic energy and epistasis). This architectural shift aligns with epistasis-driven evolution models [44], which demonstrate that RNA secondary structure constraints impose epistatic patterns shaping viral evolutionary trajectories. The electrostatic dominance suggests Omicron evolution has been constrained by the requirement to maintain favorable charge complementarity at the RBD-ACE2 interface while simultaneously disrupting antibody epitopes—a dual optimization achieved through careful selection of charged substitutions.

The complete functional reversal of Q493R and Q498R—from unfavorable (+4 kJ/mol) or neutral (+0.1 kJ/mol) in pre-Omicron to strongly favorable (−56 and −72 kJ/mol) in Omicron—represents a remarkable example of context-dependent mutation effects. DMS studies have demonstrated that N501Y, present in multiple VOCs, causes epistatic shifts in the effects of mutations at other sites, and that the availability of specific affinity-enhancing mutations changes as the RBD accumulates substitutions [33,45]. Our per-residue decomposition provides an atomistic resolution to these epistatic patterns, showing that Q493R forms a novel salt bridge with ACE2 E35 (68% occupancy in Omicron, 0% in pre-Omicron) and Q498R establishes electrostatic coupling with D38 (54% occupancy), creating an entirely new binding interface topology that compensates for the loss of the ancestral K417-D30 interaction eliminated by K417N.

The directional discrepancy between MM-PBSA–estimated binding enthalpies and experimental ACE2 affinity measurements (Pearson r = 0.682, *p* = 0.007; *n* = 14; Figure 15) is mechanistically interpretable and consistent with the known enthalpic approximation of the single-trajectory MM-PBSA protocol, which excludes entropic contributions (−TΔS). In highly mutated Omicron variants, interface restructuring generates new favorable enthalpic contacts—most notably the compensatory salt bridges Q493R-E35 (~68% MD occupancy in Omicron, 0% in pre-Omicron) and Q498R-D38 (~54% versus 0%)—that are quantified by MM-PBSA (~130 kJ/mol combined enthalpic gain). However, the concurrent loss of the dominant K417-D30 electrostatic anchor (0% Omicron occupancy versus ~100% pre-Omicron, confirmed directly by MD trajectory analysis) and the increase in interfacial conformational flexibility impose entropic penalties that reduce net binding free energy below what enthalpic calculations alone suggest. Three independent lines of evidence support this interface-reconfiguration model: (i) K417-D30 salt-bridge occupancy drops to zero in all Omicron variants in MD trajectories; (ii) Q493R-E35 and Q498R-D38 compensatory bridges are confirmed by the same MD analysis; and (iii) the MMPBSA-experimental discrepancy scales linearly with mutation burden (r = 0.927, *p* < 0.0001), consistent with progressively larger entropic costs as mutational complexity increases—a pattern predicted by enthalpy–entropy compensation theory [46,47] and consistent with reports that Omicron spike conformational dynamics differ substantially from those of ancestral strains [48,49]. Taken together, MM-PBSA is used throughout this study to characterize enthalpic interface reorganization and identify dominant energy components, within the benchmarked accuracy established by the experimental comparison.

The strong inverse correlation between B-cell epitope conservation and breakthrough-infection rates (ρ = −0.82, *p* < 0.001) provides an epidemiological validation of our computational epitope mapping. Cao et al. demonstrated that over 85% of tested neutralizing antibodies escaped by Omicron through mutations at key epitope positions K417N, G446S, E484A, and Q493R—four of the 15 RBD mutations we analyzed [50]. The 51.6% reduction in B-cell epitope conservation from pre-Omicron (74.3%) to Omicron (35.9%) quantifies this escape at the population level, integrating effects across 567 characterized B-cell epitopes. Our analysis demonstrates differential epitope targeting: B-cell epitopes showed extensive erosion, while T-cell epitopes remained largely conserved (91.5% to 87.2%; only a 4.7% relative reduction), consistent with differences in selection pressure on humoral versus cellular immunity [51,52,53]. The preservation of T-cell epitope conservation despite extensive B-cell epitope erosion has profound implications for next-generation vaccine design. CD8^+^ T cells target a broad range of epitopes from both structural and nonstructural proteins, which are often conserved across strains [54,55,56,57]. The breadth of CD8^+^ T-cell responses, together with high conservation, enables cross-recognition of viral variants. Our findings support vaccine strategies incorporating conserved T-cell epitopes, particularly from non-spike proteins, to provide cross-reactive immunity against emerging variants.

A key contribution of this work is the development of predictive computational models that enable prospective variant risk assessment. To ensure robust generalization with limited sample sizes, all models were evaluated under a two-loop nested cross-validation framework (outer leave-one-out, inner adaptive K-fold; constrained hyperparameter grids; 100 permutation tests). The Breakthrough-Infection Proxy Predictor was trained on the *n* = 30 variants with confirmed CoV-Spectrum Growth Advantage data. The optimal architecture—Tier 3, combining epitope-disruption scores with physicochemical properties (isoelectric point, GRAVY hydrophobicity index, net charge)—achieved nested CV R^2^ = 0.394 (95% CI [−0.780, 0.824], permutation *p* = 0.001, and dummy baseline R^2^ = −0.070)—demonstrating that epitope-based structural features capture genuine predictive signal for immune-escape phenotype far exceeding the permutation null distribution. For vaccine effectiveness prediction, integrated models combining epitope scores, net charge, and structural stability achieved nested CV R^2^ = 0.609 (95% CI [0.386, 0.750]; permutation *p* = 0.008; *n* = 9), with epitope disruption contributing the largest coefficient magnitude (−4.2) compared to net charge (−0.8) and structural stability (−2.1). This quantitative framework, validated under strict out-of-sample conditions, enables the identification of variants likely to compromise vaccine protection before large-scale epidemiological data become available.

Our dual-receptor-binding analysis revealed that CD147 binding operates through distinct mechanisms (hydrophobicity-dominant, interface GRAVY = −406.59) compared to ACE2 (electrostatic-dominant). Under nested cross-validation, the CD147 binding predictor achieved statistically supported out-of-sample performance (R^2^ = 0.548; 95% CI [0.257, 0.710]; permutation *p* = 0.002; *n* = 15). Importantly, sequence-only models (R^2^ = 0.130; permutation *p* = 0.040) do not require computationally expensive MD simulations, enabling the rapid screening of emerging variants within hours of sequence availability. Dual-receptor models combining ACE2 and CD147 features achieved significant booster vaccine effectiveness predictions (R^2^ = 0.609; 95% CI [0.386, 0.750]; permutation *p* = 0.008; *n* = 9), validating the utility of multi-receptor frameworks for variant characterization. Applied to eight recent Omicron sub-lineages lacking MD data (XBB.1.5.70, BA.2.86, HK.3, EG.5, CH.1.1, JN.1, JN.1.11.1, KP.3), our sequence-based models predicted moderate ACE2 binding affinities (−255 to −511 kJ/mol), consistent with a maintained cellular entry capacity despite continued epitope erosion. These predictions aligned with the observed epidemiological dominance of JN.1 and KP.3 lineages in late 2023–2024, prospectively validating the utility of this approach for variant surveillance before phenotypic data become available.

Our demonstration that Omicron employs qualitatively distinct evolutionary strategies—epistasis-dominant compensation, interface reorganization, and accelerated epitope erosion—supports the hypothesis that Omicron arose through prolonged evolution in an immunocompromised host, rather than stepwise accumulation in the general population [58,59,60]. The “chronic infection hypothesis” proposes that extended within-host replication in immunocompromised individuals allows sufficient time for the virus to accumulate and co-select mutations that would be individually deleterious but collectively beneficial. Corey et al. reported that 25% of immunocompromised individuals experience prolonged infections (≥21 days), with some lasting hundreds of days [60]. The co-residence of multiple mutations within a single host enables epistatic co-selection—mutations that reduce ACE2 affinity but enhance antibody escape can persist if accompanied by compensatory mutations, precisely the pattern we observe in Omicron.

The alternative hypothesis—that Omicron evolved in an animal reservoir—is supported by observations that Q493R, Q498R, and N501Y appear in mouse-adapted strains [61,62]. Regardless of origin, the variant employs the multi-objective optimization strategy we characterize. The closest sequences of Omicron date to mid-2020, representing over a year of unobserved evolution—consistent with either chronic infection or zoonotic circulation. The key insight is that the success of Omicron required the simultaneous optimization of three independent fitness dimensions, unlikely through sequential single-mutation accumulation but feasible through prolonged co-evolution [63,64,65].

Current genomic surveillance systems focus predominantly on individual mutations or counts of spike protein mutations as risk indicators [20]. While effective for characterizing established variants, these reactive approaches cannot anticipate which emerging lineages pose the greatest risk. Our integrated framework suggests that pandemic risk emerges from synergistic interactions across three orthogonal fitness dimensions, rather than from single mutations or linear mutation accumulation. The strong correlation between epistasis-per-mutation and mutation count (ρ = 0.90) provides a predictive indicator: Variants accumulating mutations at accelerated rates likely employ epistatic compensation and warrant priority assessment before achieving widespread circulation.

Epistatic models using Direct Coupling Analysis outperform non-epistatic approaches for predicting mutable sites [66]. Our multi-dimensional framework extends these approaches by integrating binding energetics (MMPBSA), epitope conservation (IEDB mapping), and compensation architecture (spatial and epistatic networks) into a unified risk assessment. Operationally, surveillance pipelines could flag emerging lineages showing: (1) mutation rates exceeding 5 RBD substitutions per 6-month period; (2) novel salt-bridge formation at the RBD-ACE2 interface; (3) B-cell epitope conservation below 50%; and (4) epistatic strengthening indicated by non-additive binding energy contributions. Such multi-dimensional monitoring would enable proactive, rather than reactive, variant assessment.

Immune imprinting—where prior exposures bias subsequent immune responses toward the original antigen—has complicated vaccine updates for emerging variants [67,68,69]. Boosting with heterologous spike proteins does not induce broader neutralizing antibodies; instead, responses predominantly target the parental Wuhan-Hu-1 strain. Bivalent vaccines targeting BA.4/BA.5 elicit markedly lower neutralizing antibodies against BQ.1.1 and XBB than against ancestral strains. This limitation arises partly because current vaccines focus on epitopes that Omicron has systematically eroded. The 51.6% reduction in B-cell epitope conservation means vaccine-induced antibodies targeting ancestral epitopes encounter substantially altered binding surfaces on circulating variants, reducing neutralization potency.

Several vaccine strategies emerge from our analysis. First, targeting conserved T-cell epitopes, particularly those from non-spike proteins, offers variant-resistant protection—our observation that T-cell conservation remained at 87.2% indicates that functional constraint limits escape. Multi-epitope vaccines incorporating conserved B-cell, CD4^+^, and CD8^+^ T-cell epitopes have shown protection against multiple VOCs [70,71,72]. Second, broadly neutralizing antibodies targeting conserved S2 regions—including stem helix and fusion peptide—may provide pan-coronavirus protection [73,74]. Third, our demonstration of interface plasticity suggests that vaccines that induce antibodies targeting conformational epitopes may rapidly escape through interface rewiring. Structure-guided antigen engineering to stabilize specific spike conformations could mitigate this evasion route.

Several limitations warrant consideration. First, MMPBSA calculations capture enthalpic contributions but not entropic effects, as evidenced by the paradoxical direction of computational and experimental binding measurements. The statistically significant but moderate correlation with experimental DMS data (r = 0.682, *p* = 0.007; *n* = 14) confirms that MM-PBSA captures directional binding trends and era-level differences but is not suited to absolute affinity prediction in highly mutated variants. All MM-PBSA–derived conclusions in this study are intentionally scoped to relative comparisons and mechanistic energy decomposition (electrostatic versus van der Waals components), consistent with the benchmarked performance level.

Second, our analysis included 18 variants, providing adequate statistical power for era comparisons but limiting generalization to future variants with potentially novel mechanisms. Third, molecular dynamics simulations (100 ns) capture intermediate timescale dynamics but may miss slower conformational transitions relevant to binding entropy. Fourth, epitope conservation calculations assume the equal immunological relevance of all characterized epitopes have equal immunological relevance. Immunodominance hierarchies may weight certain epitopes more heavily. Finally, the cross-sectional design cannot establish causation between evolutionary mechanisms and transmission fitness.

## 5. Conclusions

The Omicron paradox—extreme mutation accumulation with maintained fitness—resolves through recognition that this variant evolved via multi-objective optimization across three orthogonal evolutionary trajectories. Immune escape through systematic epitope erosion (ρ = −0.82) explains the selective pressure driving mutation accumulation. The interface reorganization through Q493R/Q498R gain-of-function mutations points out how binding was maintained despite the loss of ancestral contacts, and epistasis-dominant compensation (*p* = 0.0093, 2.0-fold stronger) describes how structural integrity was preserved despite destabilizing burden. These trajectories are largely independent, with weak cross-correlations supporting the simultaneous optimization of multiple fitness objectives. This evolutionary strategy represents a qualitative shift from pre-Omicron conservative accumulation, enabled by chronic infection or reservoir circulation, permitting the co-selection of epistatic mutation combinations. The integrated predictive framework we present—combining computational binding predictions (R^2^ = 0.984 for CD147, R^2^ = 0.917 for dual-receptor vaccine effectiveness), epitope mapping (R^2^ = 0.9999 for breakthrough prediction), and compensation network analysis—provides a multi-dimensional paradigm for prospective variant surveillance and risk assessment. As SARS-CoV-2 continues to evolve, deploying these predictive models for monitoring Omicron-like multi-objective optimization signatures may enable proactive identification of high-risk variants before they achieve pandemic impact.

## Data Availability

The dataset is available upon request from the corresponding author, A.N.A., or from O.A.S. All computational workflows, analysis scripts, and model code produced for this study are openly available at the project repository: https://github.com/OASolliman590/BeyondTheAbyss_SARS2 (accessed on 30 January 2026). Comprehensive documentation, environment setup instructions, and version-tagged releases are included to facilitate reproducibility. Molecular Dynamic Simulation datasets are archived on Zenodo and are accessible at https://zenodo.org/records/17780782 (accessed on 30 January 2026).

## Abbreviations

ACE2	Angiotensin-converting enzyme 2
APBS	Adaptive Poisson–Boltzmann Solver
AUC	Area under the curve
BSA	Buried surface area
CD147	Cluster of differentiation 147 (Basigin)
COVID-19	Coronavirus disease 2019
DMS	Deep mutational scanning
EMMPRIN	Extracellular matrix metalloproteinase inducer (Basigin/CD147)
GISAID	Global Initiative on Sharing All Influenza Data
GROMACS	Groningen Machine for Chemical Simulations
GRAVY	Grand average of hydropathicity
HADDOCK	High Ambiguity Driven Protein–Protein Docking
IEDB	Immune Epitope Database
LOO-CV	Leave-one-out cross-validation
MD	Molecular dynamics
MM-PBSA	Molecular mechanics Poisson–Boltzmann surface area
NPT	Isothermal–isobaric ensemble (constant number of particles, pressure, temperature)
NTD	N-terminal domain
NVT	Canonical ensemble (constant number of particles, volume, temperature)
OWID	Our World in Data
PBC	Periodic boundary conditions
PDB	Protein Data Bank
PME	Particle Mesh Ewald
RBD	Receptor-binding domain
RMSD	Root-mean-square deviation
RMSF	Root-mean-square fluctuation
ROC	Receiver operating characteristic
SARS-CoV-2	Severe acute respiratory syndrome coronavirus 2
SASA	Solvent-accessible surface area
SPC	Simple point charge (water model)
SVR	Support vector regression
VOC	Variant of concern
VMD	Visual Molecular Dynamics
WHO	World Health Organization
WT	Wild-type

## Appendix A

**Table A1:** Data sources and databases used in this study.

Data Type	Source/Database	URL/Identifier	Data Retrieved	Access Date
SARS-CoV-2 spike reference	UniProt	P0DTC2	Wild-type S-protein sequence	1 January 2024
Variant mutations	Nextstrain CoVariants	github.com/hodcroftlab/covariants	31 variant profiles	1 July 2024
Reference genome	NCBI GenBank	YP_009724390.1	Canonical Spike	15 January 2024
RBD-ACE2 template 1	RCSB PDB	6M0J	Crystal structure	15 January 2024
RBD-ACE2 template 2	RCSB PDB	6LZG	Crystal structure	15 January 2024
CD147 receptor	RCSB PDB	4U0Q (chain B)	Crystal structure	15 January 2024
B-cell epitopes	IEDB	www.iedb.org	567 RBD epitopes	1 June 2024
T-cell epitopes	IEDB	www.iedb.org	97 RBD epitopes	1 June 2024
Neutralization data	Stanford CoV-RDB	covdb.stanford.edu	Fold-reduction values	1 July 2024
DMS data	Bloom Lab	github.com/jbloomlab	ACE2 binding fitness	1 March 2024

**Table A2:** Epidemiological data sources.

Metric	Data Source	Details	Access Method
Mutational burden/Sequence divergence	Nextstrain CoVariants database	Variant-defining mutations	GitHub repository (github.com/hodcroftlab/covariants)
Variant prevalence	CoV-Spectrum/LAPIS API	Aggregates genomic surveillance from GISAID (~2.1M sequences)	REST API queries
Variant prevalence (validation)	outbreak.info	Academic consortium providing prevalence by location	API queries
Vaccination data	Our World in Data (OWID)	people_fully_vaccinated, total_boosters, new_cases_smoothed	CSV downloads
Prevalence dynamics	CoV-Spectrum/LAPIS API	Time-series global variant proportions from GISAID	REST API queries
Vaccination-era circulation	Our World in Data (OWID)	Vaccination coverage by country and time period	CSV downloads
Geographic spread	CoV-Spectrum	Number of countries with confirmed detections	LAPIS API
Case counts	Our World in Data	new_cases_smoothed by country	CSV downloads
Genomic surveillance	GISAID	Via CoV-Spectrum aggregation	Indirect access

**Table A3:** Molecular dynamics simulation parameters.

Parameter	Energy Minimization	NVT Equilibration	NPT Equilibration	Production
Algorithm	Steepest descent	Leap-frog	Leap-frog	Leap-frog
Time step (fs)	-	2	2	2
Duration	50,000 steps	100 ps	100 ps	100 ns
Temperature (K)	-	310	310	310
Pressure (bar)	-	-	1.0	1.0
Thermostat	-	V-rescale	V-rescale	V-rescale
Barostat	-	-	Parrinello–Rahman	Parrinello–Rahman
τ_T (ps)	-	0.1	0.1	0.1
τ_P (ps)	-	-	5.0	5.0
Compressibility (bar^−1^)	-	-	4.5 × 10^−5^	4.5 × 10^−5^
Cutoff scheme	Verlet	Verlet	Verlet	Verlet
Electrostatics	PME	PME	PME	PME
Real-space cutoff (nm)	1.2	1.2	1.2	1.2
vdW cutoff (nm)	1.2	1.2	1.2	1.2
Constraints	-	LINCS (h-bonds)	LINCS (h-bonds)	LINCS (h-bonds)
Force field	GROMOS54a7	GROMOS54a7	GROMOS54a7	GROMOS54a7
Water model	SPC	SPC	SPC	SPC
Ionic strength (M)	0.15	0.15	0.15	0.15

**Table A4:** Software and version information.

Software	Version	Purpose
Python	3.8+	General programming
NumPy	1.21+	Numerical operations
Pandas	1.3+	Data manipulation
SciPy	1.7+	Statistical tests
Scikit-learn	1.0+	Machine learning
Matplotlib	3.4+	Visualization
Seaborn	0.11+	Statistical visualization
NetworkX	2.8+	Graph analysis
Biopython	1.79+	Sequence handling
MDAnalysis	2.0+	Trajectory analysis
GROMACS	2023.3	MD simulations
VMD	1.9.3	Molecular visualization
PyMod	3.0	Homology modeling interface
MODELLER	10.1	Comparative modeling
HADDOCK	2.4	Protein–protein docking
g_mmpbsa	Compatible with GROMACS 2024.1	Binding energy
APBS	3.0+	Poisson–Boltzmann solver
Maestro	2021-4	Structure preparation

**Table A5:** Variant inclusion tiers and inclusion criteria.

Analysis Tier	N (Variants)	Purpose	Inclusion Criteria	Data Required	Exclusions/Notes
Full variant set	**32**	Complete panel for epitope/epidemiology/ML where inputs are available for all variants	All variants in the master dataset used for cross-era descriptive and predictive analyses	Epitope metrics + epidemiological fields (and any other globally complete features)	Use this tier only when the required inputs are 100% complete across variants
Core results set	**27**	Main mechanistic narrative and era-stratified comparisons	WHO VOC + major VOI with substantial global circulation; includes **10 pre-Omicron** (Alpha, Beta, Gamma, Delta, Epsilon, Lambda, Kappa, Eta, Iota, Mu) and **17 Omicron lineages (BA.1 through KP.3)**	Sequence-defined variant mutations + epitope mapping + epidemiological summaries	Explicitly state this tier at the start of Results and in each relevant figure caption
MD/MM-PBSA subset	**18**	Structural energetics (ΔΔG), interface contacts, decomposition, salt-bridge occupancy	Variants with completed MD trajectories and MM-PBSA calculations under a single protocol	MD trajectories + MM-PBSA outputs	List the included variants in Appendix B.1.1
External benchmark subset (DMS matched)	**14**	Experimental cross-check: computed MM-PBSA vs. experimental DMS affinity	Variants with **matched** computational (MM-PBSA) and experimental (Bloom Lab DMS) ACE2-binding measurements	Experimental DMS binding metric + matched computed MM-PBSA ΔΔG	Benchmark is explicitly limited to this subset; report “*n* = 14” in caption and text

## Appendix B. Supplementary Methods

### Appendix B.1. Sequence Analysis Pipeline

#### Appendix B.1.1. Variant Sequence Generation

The SARS-CoV-2 spike protein reference sequence (UniProt: P0DTC2, 1273 amino acids) was retrieved and used as the template for variant generation. Variant-defining mutations were obtained from the Nextstrain CoVariants GitHub repository (https://github.com/hodcroftlab/covariants, accessed on 30 January 2026), which maintains curated mutation lists for all designated variants.

Variants analyzed (*n* = 31): pre-Omicron (*n* = 10): WT, Alpha, Beta, Gamma, Delta, Epsilon, Eta, Iota, Kappa, Lambda; Omicron sub-lineages (*n* = 21): BA.1, BA.1.1, BA.2, BA.2.12.1, BA.2.75, BA.2.75.2, BA.4, BA.5, BF.7, BQ.1, BQ.1.1, XBB, XBB.1, XBB.1.5, XBB.1.16, XBB.2.3, EG.5, EG.5.1, BA.2.86, JN.1, KP.2

#### Appendix B.1.2. Physicochemical Property Calculations

Properties were computed using Bio.SeqUtils.ProtParam with the following algorithms:

Net charge at pH 7.0:

The calculation uses the Henderson–Hasselbalch equation:

$$\mathrm{pH}=pK_{a}+{log}_{10}\left(\frac{\left[A^{-}\right]}{\left[\mathrm{HA}\right]}\right)$$

Standard pK_a_ values used: N-terminus: 9.69; C-terminus: 2.34; Asp (D): 3.9; Glu (E): 4.25; His (H): 6.0; Cys (C): 8.33; Tyr (Y): 10.0; Lys (K): 10.5; Arg (R): 12.4

Net charge summation:

$$Q_{\mathrm{net}}=\sum_{i\in \mathrm{basic}}\frac{1}{1+10^{\mathrm{pH}-pK_{a,i}}}-\sum_{j\in \mathrm{acidic}}\frac{1}{1+10^{pK_{a,j}-\mathrm{pH}}}$$

GRAVY Index:

$$\mathrm{GRAVY}=\frac{1}{N}\sum_{i=1}^{N}H_{i}$$

where $H_{i}$ is the Kyte–Doolittle hydrophobicity value for residue i.

### Appendix B.2. Homology Modeling Protocol

#### Appendix B.2.1. Template Selection

Template structures were identified using BLASTp against the PDB with the following criteria: E-value < 1 × 10^−50^; sequence identity > 95%; resolution < 3.0 Å; RBD in receptor-accessible “up” configuration.

**Table A6:** Template crystal structures selected for homology modeling of the SARS-CoV-2 RBD.

PDB ID	Resolution (Å)	Description	Chain
6M0J	2.45	RBD-ACE2 complex	E (RBD)
6LZG	2.50	RBD-ACE2 complex	B (RBD)

#### Appendix B.2.2. Structure Preparation (Maestro)

1. Remove crystallographic waters and non-essential solvent molecules.
2. Complete missing side chains using Prime.
3. Add hydrogen atoms at pH 7.0.
4. Assign protonation states using PROPKA.
5. Restrained energy minimization (OPLS_2005 force field, RMSD convergence 0.30 Å).

#### Appendix B.2.3. MODELLER Parameters

Comparative modeling was performed using PyMod 3.0, a PyMOL 3.1.4.1 plugin interface for MODELLER v10.1. Target RBD sequences were aligned to the template structure (PDB: 6M0J) using ClustalOmega. For each variant, 100 models were generated using the “automodel” class with “refine_very_slow” loop refinement. Model selection was based on the minimal Discrete Optimized Protein Energy (DOPE) score and GA341 structural quality.

#### Appendix B.2.4. Model Quality Assessment

**Table A7:** Quality assessment criteria and software tools used to evaluate homology models, including energy-based, stereochemical, and global structural quality metrics for model selection.

Metric	Threshold	Tool
DOPE score	Lowest among models	MODELLER
GA341 score	>0.7	MODELLER
Ramachandran favored	>90%	PROCHECK
Ramachandran outliers	<2%	PROCHECK
QMEAN-DisCo	>0.6	SWISS-MODEL

RMSD Calculation:

$$\mathrm{RMSD}=\sqrt{\frac{1}{N}\sum_{i=1}^{N}{\left(r_{i}-r_{i}^{\mathrm{ref}}\right)}^{2}}$$

### Appendix B.3. Protein–Protein Docking Protocol (HADDOCK 2.4)

#### Appendix B.3.1. Active Residue Definition

**RBD active residues (based on crystallographic contacts):** K417, G446, Y449, Y453, L455, F456, A475, F486, N487, Y489, Q493, G496, Q498, T500, N501, G502, Y505

**ACE2 active residues:** S19, Q24, T27, F28, D30, K31, H34, E35, E37, D38, Y41, Q42, L79, M82, Y83, K353, G354, D355, R357

**RBD active residues (CD147 interface):** R403, L455, N481, V483, E484, Y489, G496, Q498, T500, N501, G502, Y505

**CD147 active residues:** T28, Q100, H102, P133, T135, D136, W137, Y140, S163, T188, K191

#### Appendix B.3.2. HADDOCK Parameters

- Solvated docking: Enabled (water as solvent).
- Flexibility: semi-flexible interface residues.
- Number of structures for rigid body docking: 1000.
- Number of structures for semi-flexible refinement: 200.
- Number of structures for water refinement: 200.
- Clustering method: FCC (Fraction of Common Contacts).
- Clustering cutoff: 0.6.

### Appendix B.4. Molecular Dynamics Simulation Parameters

#### Appendix B.4.1. System Setup

Topology was generated using “pdb2gmx” with the GROMOS54a7 force field and SPC water model. The system was solvated in a cubic box with a 1.0 nm buffer distance between the solute and the box edge (“editconf, solvate”). Neutralization was performed by adding 0.15 M NaCl ions (“genion”).

#### Appendix B.4.2. Energy Minimization (em.mdp)

**Table A8:** Energy minimization parameters used prior to equilibration of the solvated and ionized simulation systems.

Parameter	Value
Integrator	Steepest descent (steep)
Tolerance	1000.0 kJ/mol/nm
Step size	0.01 nm
Max steps	50,000
Cutoff scheme	Verlet
Electrostatics	PME (1.2 nm cutoff)
vdW interactions	Cutoff (1.2 nm)
Periodic Boundary	xyz

#### Appendix B.4.3. NVT Equilibration (nvt.mdp)

**Table A9:** NVT equilibration parameters used to stabilize system temperature at 310 K before pressure equilibration.

Parameter	Value
Integrator	md (Leap-frog)
Steps	50,000 (100 ps)
Time step	0.002 ps
Temperature coupling	V-rescale (310 K)
Coupling groups	Protein, Non-Protein
Time constant	0.1 ps
Constraints	h-bonds (LINCS, order 4)
Velocity generation	Yes (Maxwell distribution at 310 K)

#### Appendix B.4.4. NPT Equilibration (npt.mdp)

**Table A10:** NPT equilibration parameters used to stabilize system pressure and density under isotropic pressure coupling.

Parameter	Value
Integrator	md
Steps	50,000 (100 ps)
Pressure coupling	Parrinello–Rahman (isotropic)
Reference pressure	1.0 bar
Compressibility	4.5 × 10^−5^ bar^−1^
Time constant (pressure)	5.0 ps
Velocity generation	No (continuation from NVT)

#### Appendix B.4.5. Production Run (md.mdp)

**Table A11:** Production molecular dynamics simulation parameters used for the 100 ns trajectories analyzed in this study.

Parameter	Value	Description
Integrator	md	Leap-frog algorithm
nsteps	50,000,000	100 ns simulation time
dt	0.002 ps	2 fs time step
Output control	10,000 steps	Trajectory/Energy saved every 20 ps
Temperature coupling	V-rescale	Modified Berendsen thermostat
Pressure coupling	Parrinello–Rahman	Extended ensemble barostat
Constraints	h-bonds	LINCS algorithm

### Appendix B.5. Trajectory Analysis Protocol

#### Appendix B.5.1. PBC Correction

Trajectory artifacts caused by PBC were corrected using GROMACS “trjconv” utilities. The processing pipeline involved three steps: (1) making molecules whole to correct broken bonds across boundaries, (2) centering the RBD in the simulation box, and (3) performing a rotational and translational fit to the reference structure (backbone atoms) to remove global motion.

#### Appendix B.5.2. RMSD Calculation

The root-mean-square deviation (RMSD) was calculated using VMD 1.9.3 with custom TCL scripts. The trajectory was aligned to the initial frame of the RBD backbone. RMSD was computed for the backbone atoms of the RBD, the receptor (ACE2/CD147), and the full complex. Additionally, an interface RMSD was calculated for residues located within 8.0 Å of the binding partner in the initial structure, providing a focused metric for interfacial stability.

RMSF calculation:

$${\mathrm{RMSF}}_{i}=\sqrt{\frac{1}{T}\sum_{t=1}^{T}{\left(r_{i}(t)-\langle r_{i}\rangle \right)}^{2}}$$

The per-residue root-mean-square fluctuation (RMSF) was calculated for C_α_ atoms to characterize local flexibility.

#### Appendix B.5.3. Hydrogen Bond Analysis

Intermolecular hydrogen bonds were analyzed using the VMD measure h-bonds command. A hydrogen bond was defined using geometric criteria: a donor-acceptor distance cutoff of 3.5 Å and a donor-hydrogen-acceptor angle cutoff of 30°. Occupancy and lifetime were tracked across the trajectory.

#### Appendix B.5.4. Salt-Bridge Analysis

Salt bridges were identified as ionic interactions between oppositely charged residues. A salt bridge was considered formed if the distance between any oxygen atom of an acidic residue (Asp, Glu) and any nitrogen atom of a basic residue (Lys, Arg) was less than 4.0 Å. This analysis specifically monitored the stability of key electrostatic interactions at the separate-chain interface.

### Appendix B.6. MM-PBSA Calculation Parameters

#### Appendix B.6.1. g_mmpbsa Input Parameters

Calculations were performed using the “g_mmpbsa” tool [31] with the following configuration:

**Table A12:** Parameters and settings used for electrostatic potential and solvation free energy calculations.

Parameter	Value	Description
PB Solver	APBS (npbe)	Nonlinear Poisson–Boltzmann equation
Grid Spacing	0.5 Å	Fine grid resolution
$\mathrm{Solute}\;\mathrm{Dielectric}\;(\varepsilon_{in}$)	4.0	Interior dielectric constant
$\mathrm{Solvent}\;\mathrm{Dielectric}\;(\varepsilon_{out}$)	80.0	Exterior dielectric constant
Ionic Strength	0.15 M	$\mathrm{NaCl}\;(\mathrm{Radii}:\;{\mathrm{Na}}^{+}$$\;0.95\;Å,\;{\mathrm{Cl}}^{-}$ 1.81 Å)
Temperature	300 K	Simulation temperature
Non-Polar Model	SASA	$\Delta G_{np}=\gamma \cdot \mathrm{SASA}+b$
Surface Tension (γ)	0.02267 kJ⋅ ${\mathrm{mol}}^{-1}\cdot$ ${Å}^{-2}$	
Offset (b)	3.849 kJ⋅ ${\mathrm{mol}}^{-1}$	
Probe Radius	1.4 Å	Solvent probe

#### Appendix B.6.2. Binding Free-Energy Equations

Binding free energies were calculated using the single-trajectory MM-PBSA method, governed by the following equations. Entropic contributions (−TΔS) were not included (enthalpic approximation).

MM-PBSA binding free energy:

$$\Delta G_{\mathrm{bind}}=\Delta E_{\mathrm{MM}}+\Delta G_{\mathrm{polar}}+\Delta G_{\mathrm{apolar}}$$

where:

$$\Delta E_{\mathrm{MM}}=\Delta E_{\mathrm{vdW}}+\Delta E_{\mathrm{elec}}$$

Poisson–Boltzmann equation:

$$\nabla \cdot \left[\varepsilon (r)\nabla \phi (r)\right]=-4\pi \rho (r)-4\pi \sum_{i}c_{i}q_{i}\lambda (r)exp\left(-\frac{q_{i}\phi (r)}{k_{B}T}\right)$$

Non-polar solvation (SASA):

$$\Delta G_{\mathrm{apolar}}=\gamma \cdot \mathrm{SASA}+b$$

#### Appendix B.6.3. Execution

Binding free-energy calculations were executed using the “g_mmpbsa” tool with the single-trajectory protocol. A representative execution command is:

g_mmpbsa -f final_noPBC.xtc -s step5_production.tpr -n index.ndx -pdie 4 -decomp -polar -apolar.

where -pdie 4 defines the solute dielectric constant, -decomp enables per-residue energy decomposition for interaction profiling, and -polar/-apolar trigger the respective solvation energy calculations defined in Appendix B.6.2.

### Appendix B.7. Epidemiological Data Processing

#### Appendix B.7.1. Data Source Overview

**Metric**	**Data Source**	**Details**
Mutational burden/Sequence divergence	Nextstrain CoVariants database	Variant-defining mutations from GitHub repository (github.com/hodcroftlab/covariants)
Variant prevalence	CoV-Spectrum/LAPIS API	Aggregates genomic surveillance from GISAID (~2.1M sequences)
Variant prevalence (validation)	outbreak.info	Academic consortium providing prevalence by location
Vaccination data	Our World in Data (OWID)	people_fully_vaccinated, total_boosters, new_cases_smoothed
Prevalence dynamics	CoV-Spectrum/LAPIS API	Variant-specific sequence prevalence and growth-advantage estimates. Variant sequences were queried by Pango lineage with a minimum proportion threshold of 10% (minProportion = 0.10) to exclude low-confidence classifications. Data were retrieved at biweekly granularity globally.
Vaccination-era circulation	Our World in Data (OWID)	Fully vaccinated persons per 100 population (people_fully_vaccinated) and total booster doses administered (total_boosters) by country and date. OWID vaccination reporting was discontinued in May 2023; see Appendix B.7.4 for missingness handling. new_cases_smoothed (7-day rolling average) to anchor denominator normalization. All raw source files are archived in the project repository under 1-CoV-Spectrum Epidemiological Data Pipeline Reference
Geographic spread	CoV-Spectrum	Number of countries with confirmed detections

#### Appendix B.7.2. CoV-Spectrum LAPIS API Queries

Variant surveillance data was retrieved programmatically from the CoV-Spectrum LAPIS API (https://lapis.cov-spectrum.org/open/v2, accessed on 30 January 2026). The extraction pipeline utilized the following query structure:

1. Mutation Extraction (Endpoint:/sample/nucleotideMutations): Parameters: variant (Pango Lineage, e.g., “BA.2.86”), dateFrom/dateTo (dominance period), minProportion (0.1). Purpose: Identifying consensus RBD mutations for structural modeling.
2. Prevalence Tracking (Endpoint:/aggregated): Parameters: variant, location (Region/Country), granularity (“month”). Purpose: Calculating growth-advantage and fitness metrics.

Example query: GET https://lapis.cov-spectrum.org/open/v2/aggregated?variant=XBB.1.5&fields=date,proportion&location=World, accessed on 30 January 2026

#### Appendix B.7.3. Growth-Advantage Calculation

Logistic growth model:

$$f(t)=\frac{f_{0}\cdot e^{st}}{1+f_{0}\cdot \left(e^{st}-1\right)}$$

where $f_{0}$ is the initial frequency, s is the selection coefficient (growth advantage per day), and t is time.

#### Appendix B.7.4. Breakthrough Proxy Calculation

The breakthrough-infection proxy quantifies the relative infection burden in the fully vaccinated population, normalized per 100,000 vaccinated individuals, as a real-world signal of vaccine escape. It was computed per variant, per country, per biweekly period using the following formula:

$${\mathrm{Proxy}}_{\mathrm{breakthrough}}=\frac{\mathrm{new}\_\mathrm{cases}\_\mathrm{smoothed}\times \mathrm{variant}\_\mathrm{prevalence}}{\mathrm{people}\_\mathrm{fully}\_\mathrm{vaccinated}}\cdot 100,000$$

where:

variant_prevalence = fraction of sequences assigned to the variant in CoV-Spectrum during that period (biweekly consensus)

new_cases_smoothed = 7-day rolling average of new COVID-19 cases from OWID

people_fully_vaccinated = total persons with completed primary series from OWID

An analogous proxy_per_100k_boosted was computed using total_boosters in the denominator.

Quality weighting and filtering: Each country–period record was assigned a quality_weight proportional to the number of sequences in CoV-Spectrum for that period (range: 0.05–1.0). Only records with quality_weight ≥ 0.70 were retained for per-variant aggregation (relaxed to ≥0.50 for the booster cohort where coverage was limited). Records with physically implausible ratios (proxy_boosted/proxy_vax < 0.05 or > 5.0) were excluded as outliers.

Aggregation: Per-variant breakthrough proxy values reported in the manuscript are the unweighted mean of qualified country–period records within each variant’s dominant circulation window, with mean ± SD across records. The number of qualifying records per variant ranged from 8 to 61 (median: 30).

Missingness handling (post-May 2023): OWID discontinued routine vaccination reporting in May 2023 (https://ourworldindata.org/). For variants circulating primarily after this date (XBB.1.5.70, BA.2.86, HK.3, EG.5, CH.1.1, JN.1, JN.1.11.1, KP.3), country-level vaccination coverage was forward-imputed using the last available OWID entry for each country (flat extrapolation under the assumption that near-saturation vaccination rates changed minimally over this period). This assumption is conservative—if actual vaccination rates declined after May 2023, the proxy denominator is overestimated, meaning the breakthrough proxy values for these variants are lower-bound estimates of true vaccine escape. These eight recent variants were, therefore, excluded from the nested-CV ML model and used only for prospective surveillance prediction (Section 3.3). All interpolation flags are retained in the raw pipeline output (vax_data_available column) for full auditability.

Note: OWID discontinued vaccination reporting in May 2023; analyses after this date use interpolated estimates.

### Appendix B.8. Epitope Conservation Analysis

#### Appendix B.8.1. IEDB Query Parameters

B-cell epitopes: Organism: SARS-CoV-2; Antigen: Spike protein; Epitope structure: Linear; Host: *Homo sapiens*; Assay type: B-cell; Qualitative measure: Positive; Immunization: Administration in vivo (excluding disease state)

T-cell epitopes: Same filters plus: MHC restriction (Class I or Class II)

#### Appendix B.8.2. Epitope Filtering

Epitopes were filtered from the Immune Epitope Database (IEDB) to ensure high-confidence, biologically relevant targets. The following exclusion criteria were applied:

Assay Type: B-cell and T-cell assays (MHC Ligand/Elution excluded).

Host Organism: *Homo sapiens* only.

Immunization Context: “Administration in vivo” (Vaccine-induced responses only); Natural infection and disease-state epitopes were excluded to model vaccine escape specifically.

Assay Outcome: “Positive” qualitative results only.

Epitope Length: ≥8 amino acids.

Sequence Mapping: Epitopes were required to map with 100% identity to the Wuhan-Hu-1 reference RBD (residues 333–527) to allow precise mutation impact assessment.

#### Appendix B.8.3. Immunodominance Weights

**Table A13:** Immunodominance weighting scheme applied to the three RBD subregions, including residue boundaries, assigned weights, and the immunological rationale used for weighted epitope conservation analyses.

Region	Residues	Weight	Rationale
RBD-1	333–363	0.85	NTD-proximal, class 2 antibodies
RBD-2	364–437	0.72	Core RBD, structural stability
RBD-3	438–527	0.90	RBM, class 1 neutralizing antibodies

#### Appendix B.8.4. Conservation Equations

Immunodominance-Weighted Conservation:

$${\mathrm{Conservation}}_{\mathrm{adjusted}}=0.7\times {\mathrm{Conservation}}_{\mathrm{weighted}}+0.3\times {\mathrm{MD}}_{\mathrm{normalized}}$$

Epitope-Disruption Score:

$${\mathrm{Disruption}}_{\mathrm{epitope}}=\frac{\sum_{i\in \mathrm{epitope}}𝟙({\mathrm{mutated}}_{i})\times w_{i}}{\sum_{i\in \mathrm{epitope}}w_{i}}$$

### Appendix B.9. Machine Learning Model Development

#### Appendix B.9.1. Feature Engineering

Molecular dynamics features: RBD backbone RMSD (mean, std); Interface RMSD (mean, std); Hydrogen bond count (mean); Salt-bridge count (mean); Buried surface area (mean).

MM-PBSA features: Total binding energy (ΔG_bind); van der Waals component (ΔE_vdW); electrostatic component (ΔE_elec); polar solvation (ΔG_polar); non-polar solvation (ΔG_apolar).

Sequence features: Net charge at pH 7.0; GRAVY index; isoelectric point; molecular weight; mutation count.

#### Appendix B.9.2. Model Training

Ridge Regression Objective:

$$L(\beta )=\sum_{i=1}^{n}{\left(y_{i}-x_{i}^{T}\beta \right)}^{2}+\alpha \sum_{j=1}^{p}\beta_{j}^{2}$$

Leave-One-Out Cross-Validation Error:

$${\mathrm{CV}}_{\mathrm{LOO}}=\frac{1}{n}\sum_{i=1}^{n}{\left(y_{i}-{\hat{y}}_{\left(-i\right)}\right)}^{2}$$

#### Appendix B.9.3. Hyperparameter Optimization

Alpha values tested: [0.001, 0.01, 0.1, 1.0, 10.0, 100.0], selection criterion: Minimum LOO-CV MSE

### Appendix B.10. Compensation Network Analysis

#### Appendix B.10.1. Residue Classification

Destabilizing Residue Criteria:

$$\mathrm{Destabilizing}:\;{\mathrm{RMSF}}_{i}>\mu_{\mathrm{RMSF}}+\sigma_{\mathrm{RMSF}}\;\mathrm{OR}\;\Delta G_{i}>+2\;\mathrm{kJ}/\mathrm{mol}$$

Stabilizing Residue Criteria:

$$\mathrm{Stabilizing}:\;{\mathrm{RMSF}}_{i}<\mu_{\mathrm{RMSF}}-\sigma_{\mathrm{RMSF}}\;\mathrm{AND}\;\Delta G_{i}<-2\;\mathrm{kJ}/\mathrm{mol}$$

#### Appendix B.10.2. Spatial Compensation

Spatial compensation was evaluated by analyzing the proximity of stabilizing residues to destabilizing sites. A compensatory pair was defined as a destabilizing residue (as classified in Appendix B.10.1) located within a Euclidean distance of 10.0 Å from at least one stabilizing residue, permitting local enthalpic rescue of the destabilized site.

#### Appendix B.10.3. Epistatic Interaction

$$\Delta \Delta G_{\mathrm{epistasis}}=\Delta \Delta G_{\mathrm{observed}}-\Delta \Delta G_{\mathrm{expected}}$$

where:

$$\Delta \Delta G_{\mathrm{expected}}=\sum_{i}\Delta \Delta G_{\mathrm{single},i}$$

#### Appendix B.10.4. Network Analysis

Compensatory networks were constructed to visualize protection mechanisms. In these graphs, nodes represented residues, and edges represented identified spatial compensation pairs (<10 Å). Network metrics, including node degree and compensation fraction (the percentage of destabilizing nodes connected to at least one stabilizing partner), were computed to quantify the robustness of the structural compensation strategy for each variant.
